# Integrative genomics of microglia implicates DLG4 (PSD95) in the white matter development of preterm infants

**DOI:** 10.1038/s41467-017-00422-w

**Published:** 2017-09-05

**Authors:** Michelle L. Krishnan, Juliette Van Steenwinckel, Anne-Laure Schang, Jun Yan, Johanna Arnadottir, Tifenn Le Charpentier, Zsolt Csaba, Pascal Dournaud, Sara Cipriani, Constance Auvynet, Luigi Titomanlio, Julien Pansiot, Gareth Ball, James P. Boardman, Andrew J. Walley, Alka Saxena, Ghazala Mirza, Bobbi Fleiss, A. David Edwards, Enrico Petretto, Pierre Gressens

**Affiliations:** 1 Centre for the Developing Brain, Department of Perinatal Imaging and Health, Division of Imaging Sciences and Biomedical Engineering, King’s College London, King’s Health Partners, St. Thomas’ Hospital, London, SE1 7EH UK; 2 PROTECT, INSERM, Université Paris Diderot, Sorbonne Paris Cité, Paris, 75014 France; 3 PremUP, F-75006 Paris, France; 4 Pierre and Marie Curie University, UMRS-1135, Sorbonne Paris Cité, F-75006 Paris, France; 5 Medical Research Council/University of Edinburgh Centre for Reproductive Health, Edinburgh, EH16 4TJ UK; 6 Cell Biology and Genetics Research Centre, St. George’s University of London, London, SW17 0RE UK; 7 Genomics Core Facility, NIHR Biomedical Research Centre, Guy’s and St. Thomas’ NHS Foundation Trust, London, SE1 9RT UK; 8 Department of Clinical and Experimental Epilepsy, UCL Institute of Neurology, London, WC1N 3BG UK; 9 Epilepsy Society, Chalfont-St-Peter, Bucks, SL9 0RJ UK; 10 Duke-NUS Medical School, 8 College Road, Singapore, 169857 Singapore

## Abstract

Preterm birth places infants in an adverse environment that leads to abnormal brain development and cerebral injury through a poorly understood mechanism known to involve neuroinflammation. In this study, we integrate human and mouse molecular and neuroimaging data to investigate the role of microglia in preterm white matter damage. Using a mouse model where encephalopathy of prematurity is induced by systemic interleukin-1β administration, we undertake gene network analysis of the microglial transcriptomic response to injury, extend this by analysis of protein-protein interactions, transcription factors and human brain gene expression, and translate findings to living infants using imaging genomics. We show that DLG4 (PSD95) protein is synthesised by microglia in immature mouse and human, developmentally regulated, and modulated by inflammation; DLG4 is a hub protein in the microglial inflammatory response; and genetic variation in *DLG4* is associated with structural differences in the preterm infant brain. *DLG4* is thus apparently involved in brain development and impacts inter-individual susceptibility to injury after preterm birth.

## Abstract (web summary)

Inflammation mediated by microglia plays a key role in brain injury associated with preterm birth, but little is known about the microglial response in preterm infants. Here, the authors integrate molecular and imaging data from animal models and preterm infants, and find that microglial expression of DLG4 plays a role.

## Introduction

The majority of preterm infants develop encephalopathy of prematurity characterised by oligodendrocyte maturation arrest, hypomyelination and reduced brain growth^1–3^. This is associated in over 30% of infants with long-term neurocognitive problems^4, 5^, and has characteristic correlates on magnetic resonance imaging (MRI) and diffusion MRI (d-MRI)^6, 7^. It is strongly associated with systemic and cerebral inflammation, with the prominent involvement of microglia^2, 8–10^.

Microglia are found preferentially within the developing white matter^2, 11^ and have both pro-inflammatory and restorative functions that are essential for normal brain development^12, 13^. However, the microglial response to preterm birth is poorly understood and a better understanding of microglial function in this context could allow therapeutic modulation to mitigate the brain injury of prematurity and other cerebral inflammatory conditions.

We have used integrative genomics to investigate the role of microglia in preterm brain development, employing a clinically relevant mouse model of interleukin-1β (IL1B)-induced systemic inflammation that recapitulates the essential features of the encephalopathy of prematurity^14^, integrating microglial-specific data from this model with ex vivo and in vitro experiments, analysis of human microglia and imaging-genomic data from preterm infants.

We now report: (a) endogenous expression of DLG4 (PSD95) by microglia in early development, which is modulated by developmental stage and inflammation; (b) a role for DLG4 as a hub protein in the microglial inflammatory response; and (c) an association between genetic variability in *DLG4* and white matter structure in the preterm neonatal brain.

## Results

### Global effects of IL1B on mouse microglial transcriptome

**Fig. 1:**
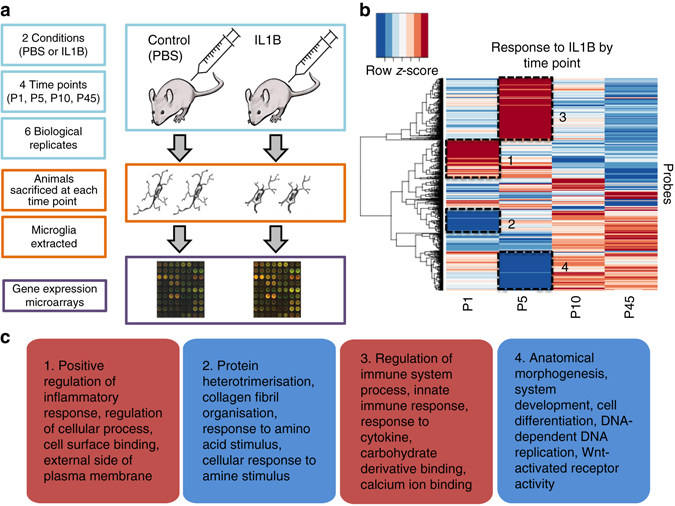
Overview of in vivo mouse model, and clustering of expression responses to IL1B by time point, with functional annotation summary. **a** IL1B mouse model experimental set-up. **b** Clustering of expression profiles in response to IL1B, showing up- and downregulated clusters at each time point (Student’s *t* test, *p* < 0.05, FDR 10%). **c** Summary functional annotation of four main clusters identified in **b**. *Red* = upregulated in IL1B vs. PBS, *Blue* = downregulated in IL1B vs. PBS. Mouse illustration: “Drawing of a grey mouse”, Author Jan Gillbank, licence CC-BY-3.0. Syringe icon made by Freepik from www.flaticon.com

In a previously validated mouse model of encephalopathy of prematurity in which IL1B is administered intra-peritoneally from postnatal days 1–5^14^ (Fig. 1a), we verified that microglia were the predominant myeloid cell in the brain (Supplementary Fig. 1a, b) and that the blood-brain barrier remained appreciably intact, with increased expression of tight junction and adherens genes (Supplementary Fig. 2a-d). P5 is comparable with human gestational age around 32 weeks^15^. CD11B+ cells were then isolated by magnetic-activated cell sorting (MACS) at postnatal days 1, 5, 10 and 45, and fluorescence-activated cell sorting (FACS) and qPCR analysis used to demonstrate these cells to be >95% pure microglia (Supplementary Fig. 1c–e). These isolated ex vivo microglia were then used for microarray gene expression analysis.

We examined the effects of systemic IL1B on the microglial transcriptome by: (a) comparing systemic IL1B exposure with control conditions (“IL1B”); (b) examining transcriptional changes over time (“Development”) and; (c) assessing whether there is a different transcriptional response to IL1B as a function of time (“Interaction”). Development captured development of microglia themselves as well as systemic maturation, and the interaction assessed whether IL1B effects were time dependent.

After accounting for multiple testing, we found thousands of genes with altered expression in each of the three responses (Supplementary Data 1). Functional enrichment analysis of the differentially expressed genes implicated numerous biological processes and pathways (Supplementary Fig. 3 and Supplementary Data 2 and 3). IL1B and Development showed some interdependency, with over-representation of cytokine-cytokine receptor interaction, transmembrane signalling and cell adhesion molecules. Using REVIGO^16^, we defined a set of representative gene ontology (GO) processes common to IL1B and Development, including immune system processes, cell adhesion, morphogenesis, regulation of multicellular organismal process and cell surface receptor signalling (Supplementary Data 4). IL1B exposure during development thus has a pervasive effect on the microglial transcriptome, engaging pathways relevant to both immune function and growth.

### Patterns of microglia gene expression response to IL1B

When expression profiles were clustered by their response to IL1B at each time point, several gene clusters became apparent (Fig. 1b and Supplementary Fig. 4), with the most prominent clusters observed at P1 and P5 (Z-score above 1 or below −1, difference between IL1B and control *p* < 0.05, false discovery rate (FDR) = 10%, two-sample Welch *t*-statistics, adjusted with Benjamini and Hochberg FDR controlling procedure). Functional annotation (Fig. 1c and Supplementary Data 5) revealed inflammatory genes upregulated at P1 and P5 in two distinct waves: an immediate response at P1 and a subsequent early response at P5. In contrast, processes related to anatomical development and DNA replication were downregulated at P1, and cell structure and binding-related processes were downregulated at P5. This transcriptional pattern was neutralised by P10 with suggestion of a reversal by P45, which was not quantified further. Systemic IL1B thus induces a rapid transcriptional response in microglia that prioritises inflammatory functions over growth.

### Gene co-expression network analysis

**Fig. 2:**
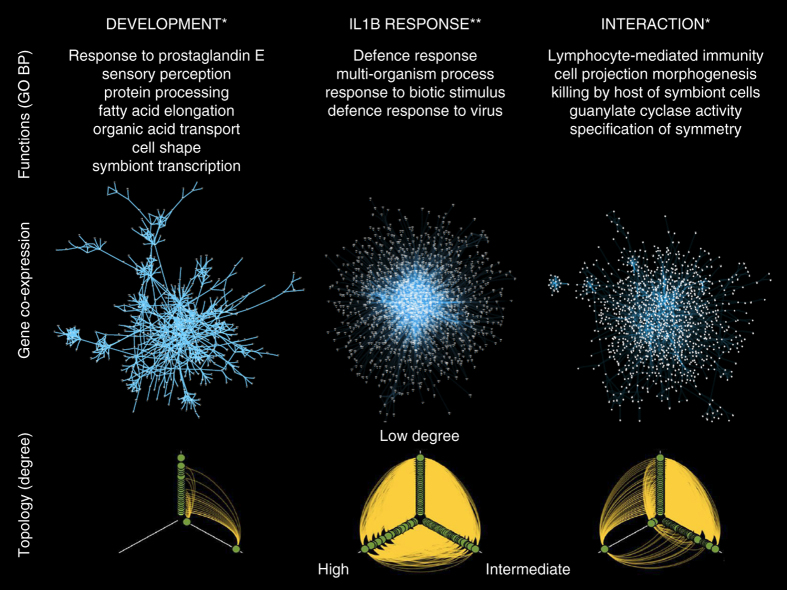
Gene co-expression networks for three responses. Development, IL1B and interaction. *Top*: summary of functional annotation. Hypergeometric test, * = nominal *p* < 0.05, ** = adjusted *p* < 0.05. *Middle*: Gene co-expression networks; nodes = genes, edges = partial correlations, local FDR = 1x10^−13^. *Bottom*: Hiveplots for each network illustrating differences in topology between responses, where nodes are arranged on axes according to their degree; axes range clockwise from top: degree < 30, 30 ≤ degree ≤ 80, degree *x* > 80

Gene and protein network-based analysis uncovers processes involved in disease^17^, with topological measures being informative of underlying biology^18^. The functionally coherent gene clusters suggested gene co-regulation, and an analysis using graphical Gaussian models^19^ revealed characteristic co-expression networks emerging in response to IL1B (IL1B), over time (Development), or with a differential response to IL1B over time (Interaction) (Fig. 2).

The three networks showed distinct gene membership and function, with only 22 genes in common (Supplementary Table 1), and notable topological differences (Supplementary Table 2). The Development network had small world topology with a high clustering coefficient and degree exponent close to 2. In contrast, the IL1B network was bigger and more homogenous, and although there were genes with high degree (i.e., hub genes with many other genes connected to them) this did not result in the formation of obvious subclusters. The Interaction network has a topological structure intermediate to the other two networks (Fig. 2, *lower panel*, and Supplementary Movie 1). This suggests that IL1B disrupts the normal small world topology, leading to a transcriptional response akin to the genomic storm previously noted in human leukocytes following severe inflammatory stress^20^.

Functional enrichment analysis showed that differences in network topology were paralleled by different biological processes and pathways (Supplementary Fig. 5 and Supplementary Data 6), with no overlap between the annotation categories (GO terms or Kyoto Encyclopedia of Genes and Genomes (KEGG) pathways) for IL1B and Development networks. The IL1B network was predominantly enriched for GO terms related to defence response, transmembrane signalling and channel activity. The Development and Interaction networks encompassed broad functional categories, spreading the networks’ genes among many different categories, so that enrichment results rarely survive multiple testing correction; nominally significant functional enrichment terms are therefore included for the annotation of the Development and Interaction networks (Fig. 2, *top panel*). This annotation implies that differences between gene networks are reflected at the functional level, with IL1B activating immune responses, while Development and Interaction have broader biological functions.

### Protein-protein interactions and neuropsychiatric genes

**Fig. 3:**
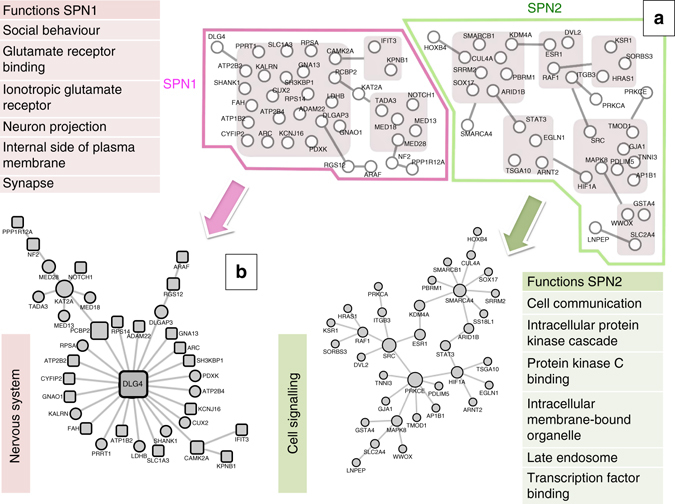
Protein-protein interactions derived from gene co-expression networks, with grouping into super-power nodes (SPNs) and functional annotation. **a** Two SPNs in power graph analysis, showing SPN1 and SPN2 members; nodes = proteins, edges = high confidence curated interactions. Proteins inside *grey boxes* form modules. Proteins connected to the outline of a *grey box* are connected to every protein inside that box. **b** Conventional visualisation of SPNs. Proteins with *square outlines* are predicted transcriptional targets of STAT3 transcription factor at the gene level. Annotation *text boxes* show summary of significant functional enrichment annotation of SPNs (hypergeometric test, adjusted *p* value <0.01)

We asked whether these co-expression relationships are conserved at the protein level and relevant to neuropsychiatric disorders linked to brain development and prematurity. The genes from all three co-expression networks were combined and their shared interactome investigated by searching for known protein-protein interactions (PPI) to highlight functional interactions, an approach previously shown to be useful for disease gene prioritisation^21^. We used a curated data set to build a robust PPI network^22^ and then carried out a power graph analysis (PGA)^23^ to identify simplified coherent PPI networks. Power graphs are lossless representations of graphs based on power nodes (sets of nodes brought together) and power edges (connecting two power nodes, so that all the nodes in the first power node are connected to all the nodes in the second power node). To further rationalise the power graph structures, we introduce the term super-power node (SPN) to refer to a set of power nodes that form a connected graph. When applied to all genes present in the three co-expression networks, we identified high-confidence connections between 96 proteins. The PGA revealed that 71 of these proteins belong to either one of two main SPNs: SPN1 or SPN2 (Fig. 3 and Supplementary Fig. 6). To corroborate the identification of these two SPNs independently, we used a separate approach (DAPPLE)^24^, which interrogates a large database of experimentally derived PPI to assess the physical connections among proteins encoded by the genes of interest. In this case, the significance of the PPI derived from the input gene list is assessed empirically. This analysis replicated the identification of 23/46 edges from SPN1 and 36/41 edges from SPN2, providing empirical support for the significance of these PPI (permutation-based *p* value <0.001, Supplementary Fig. 7).

PPIs have rarely been measured in the context of distinct cell types, tissues or in specific disease conditions, making it challenging to model and understand context-related phenotypes, but it has been shown that heterogeneous genomic data contain functional information of protein-DNA, protein-RNA, protein-protein and metabolite-protein interactions^25, 26^. We therefore sought additional external validation of cell-type specificity for SPN1 and SPN2 by querying the GIANT database of tissue-specific gene networks that includes mapping to tissue and cell-lineage-specific functional contexts^17^. Glia-specific gene interactions within SPN1 and SPN2 were reconstructed with high confidence from this prior experimental data (Supplementary Fig. 8a), further supporting the consistency and specificity of our findings.

Genes coding proteins in the SPNs came from all three gene networks with minimal overlap (Supplementary Fig. 9), suggesting different transcriptional networks (Fig. 2) might converge to less redundant structures at the protein level (Fig. 3), probably reflecting the known modular architecture of PPI networks^27^ reflected in the identification of SPN1 and SPN2. Functional annotation of the two SPNs indicated that these are distinct in function, with SPN1 being significantly enriched for proteins involved in nervous system processes and SPN2 for cell signalling and transcriptional regulation (Fig. 3 and Supplementary Table 3). Consistent with this, SPN1 showed tissue expression specific to the brain, whereas SPN2 had a broader tissue distribution (Supplementary Data 7 and 8).

We tested whether SPN members were enriched for genes involved in brain disorders. The gene disease annotation tool^28^ showed that SPN1 is significantly and specifically enriched for autism and schizophrenia (*p* < 0.05, 10,000 permutations^28^) (Supplementary Data 9). SPN2 has a broader though similar enrichment within the psychology and psychiatry category (Supplementary Data 9), alongside a very general enrichment across all systems (Supplementary Data 10), implying an important but less specific role in brain disorders. The WebGestalt tool independently showed specific enrichment in SPN1 for genes involved in brain disease^29^ (hypergeometric test, adjusted *p* < 0.05, Supplementary Table 4).

These analyses suggest that the gene co-expression relationships observed in microglial cells after in vivo IL1B treatment are at least in part conserved at the protein level, and can be synthetised by two major PPI modules, which represent functional modules in PPI networks. These are functionally distinct, with SPN1 specifically enriched for genes involved in neuropsychiatric disorders linked to prematurity, such as *DLG4* in schizophrenia and autism^30–32^, *SHANK1* in autism^33^ and *CAMK2A* in several phenotypes^34^.

### Transcriptional regulation of SPNs

We examined the potential regulation of SPN1 and SPN2 by transcription factors (TFs) and searched for potential regulatory relationships mediated by TFs, which can determine coordinated expression of several target genes.

Analysis of transcription factor-binding site (TFBS) motifs using the PASTAA algorithm^35^ indicated that an SPN2 member, STAT3 (signal transducer and activator of transcription 3), as well as other members of the signal transducers and activators of transcription (STAT) family of TFs (STAT6 and STAT1-alpha), are significantly predicted to bind the promoters of 22/36 (61%) members of SPN1 (hypergeometric test, *p* < 0.05, Supplementary Tables 5–7). The link between the STAT family of TFs and genes in SPN1 was also supported by an independent analysis using the gene set enrichment analysis tool in the molecular signatures database (Broad Institute)^36^ to assess the overlap between 615 gene sets (containing genes that share a common TFBS) and the genes in SPN1/2. This showed a significant association of members of SPN1 (*Med13*, *Kcnj16* and *Ifit3*) with STAT1 and STAT2 TFs (FDR = 2.8%), further supporting a link between SPN2 and SPN1 via the STAT family of TFs.

To seek additional experimental support for the predicted interaction between SPN1 members and the STATs TFs, we investigated the genomic distribution of binding sites of the family of signal transducers and activators of transcription (STAT1, STAT3 and STAT5) in a data set of lipopolysaccharide (LPS)-stimulated primary microglial cultures^37^. Chromatin immunoprecipitation-promoter microarray data (ChIP-Chip) indicated STAT binding at the promoters of 8/35 (22%) of the genes in SPN1 with any of STAT1, STAT3 or STAT5 (Supplementary Table 8). On inspection of the STAT3 TF gene expression response to IL1B exposure (Supplementary Fig. 10a), its mRNA level was found to be significantly higher at P1 in microglia exposed to IL1B vs. controls, and significantly lower at P5 (FDR = 0.1%). At P1, the mRNA levels of the 22 predicted transcriptional targets of STATs in SPN1 vary, with log_2_ ratios in the range −1.02 to 1.24 (Supplementary Fig. 10b). These results support a potential link between SPN2 and SPN1 (Supplementary Fig. 10c), and suggest that STAT3 TF in SPN2 may regulate SPN1, perhaps through early *Stat3* gene activation in IL1B-exposed cells, as previously reported^38^.

### Confirmation of effects of STAT3 TF on SPN members

Using CD11B+ cells MACS-isolated at P1 and exposed to a vehicle solution or IL1B + IFNg (chosen because they are highly regulated in the brains of IL1B exposed mice (Gressens, unpublished data) and cause a moderate but consistent inflammatory reaction in vitro^39, 40^), we tested the transcriptional role of STAT3 in the interaction between SPN1 and SPN2 by STAT3 pharmacological inhibition followed by RT-qPCR. MACS-isolated microglia were stimulated by IL1B + IFNg and exposed to vehicle or a small molecule inhibitor of STAT3 (BP-1-102) that binds to the three subpockets of STAT3 SH2 domain and blocks STAT3 phosphorylation, dimerization and DNA-binding activity. Gene expression for a set of genes representing general microglia function markers and a subset of genes from SPN1 and SPN2 was measured by qPCR (Supplementary Table 13). The profiles and features of the markers were previously characterised by us with in vitro microglia^39^. Given the high purity of the MACS-isolated CD11B+ cells (>95% microglia, Supplementary Fig. 1), we employ them as markers of general microglia activity states and a means of assessing the potential role of the cell rather than for the purposes of cell-type specificity. We used 11 markers broadly grouped as markers of classic pro-inflammatory actions (*Ptgs2, Cd32, Cd86* and *Nos2*), immunomodulatory markers (*Il1rn, Il4ra, Socs3* and *Sphk1*) and regenerative function markers (*Lgals3, Igf1* and *Cd206*)^39^. IL1B + IFNg induced expression of several pro-inflammatory and immunomodulatory markers and repressed expression of regenerative markers as expected. STAT3 inhibition decreased expression of three of the four pro-inflammatory markers (*Cd32*, *Cd86* and *Nos2*), but had no significant effect on the repression of regenerative markers. By contrast, IL1B + IFNg reduced expression of immunoregulatory marker *Socs3*, while *Sphk1* expression was increased by STAT3 inhibition (Supplementary Fig. 11).

Regarding the analysis of genes from SPN1 (*Dlg4* and *Notch1*), we found no effect on *Dlg4* of inflammation or STAT3 inhibition. This lack of response of *Dlg4* gene expression to IL1B is supported by the microarray measurements at P1, which show no significant difference between IL1B and control (PBS) (Supplementary Fig. 10, Student’s *t* test, *p* = 0.32). However, expression of DLG4 protein in neurons is strongly regulated by post-translational modifications, and these data may not provide reliable information on changes at the protein level. *Notch1* gene expression was also unaffected by either inflammation or STAT3 inhibition. For the analysis of *SPN2* genes, we observed that IL1B + IFNg exposure significantly increased expression of *Stat3*, and STAT3 inhibition significantly decreased *Arnt2* and *Hif1a* (Supplementary Fig. 11).

Taken together, these data confirm that IL1B + IFNg exposure induces both pro-inflammatory and immunomodulatory microglial responses, and that STAT3 presence is required for the pro-inflammatory response and typical modulatory component to occur. The transcriptional relationship between SPN1 and SPN2 in the context of inflammation is less clear, and possibly post-transcriptional.

### DLG4 is central to SPN1 and has a novel role in microglia

To clarify how the systemic IL1B inflammatory stimulus leads to specific neuronal and neurological effects, we focused on DLG4, a member of the membrane-associated guanylate kinase family. DLG4 has not previously been known to have a function in microglia, but is the hub protein of SPN1 (Fig. 3), and has an established neurodevelopmental role in synaptic plasticity in neurons^41^. Our data suggest the hypothesis that DLG4 plays a role in the brain’s response to inflammation and may be a potential link between inflammation and both preterm brain injury and neuropsychiatric disease.

### DLG4 at the microglial membrane is dynamically modulated

**Fig. 4:**
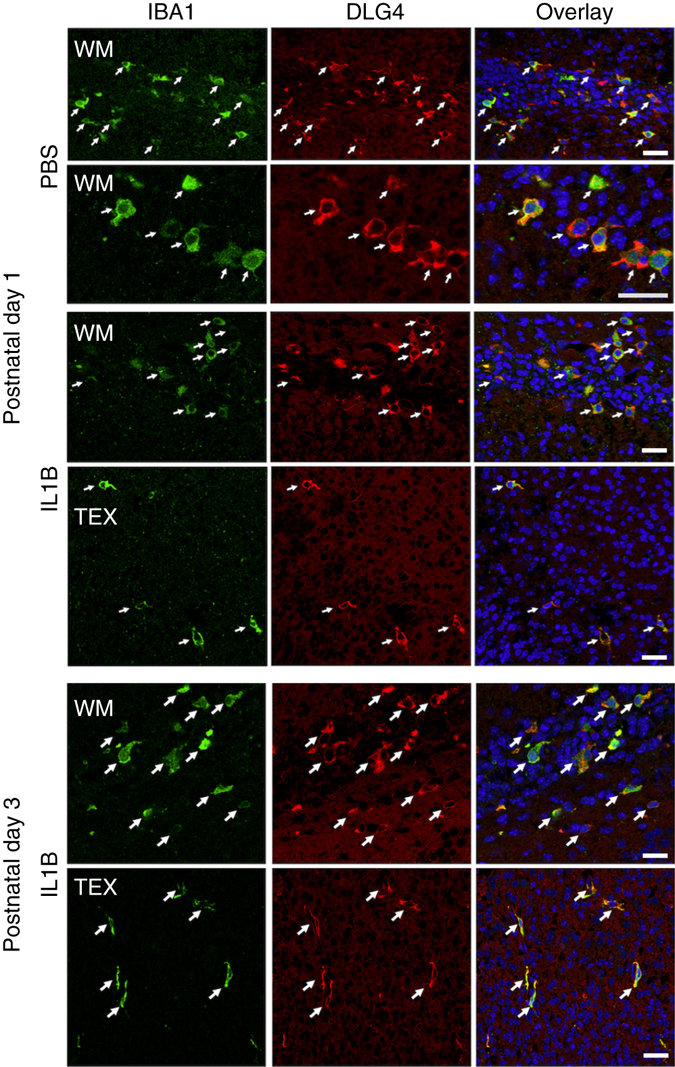
Effects of exposure to neuroinflammation on DLG4 expression in mouse microglia in vivo. Double labelling of MACS-isolated mouse microglia (IBA1+) and DLG4 (PSD95) under control (PBS) and inflammatory (IL1B) conditions in the subcortical white matter (WM) and sensorimotor cortex (TEX). IBA1 and DLG4 were exclusively co-localised (*arrows*) at P1 in both PBS and IL1B mice but at P3 IBA1 and DLG4 co-localisation (*arrows*) was observed only in IL1B mice, not in control (PBS) mice. *Scale bars* = 30 µm

We investigated whether DLG4 protein could be observed in mouse microglia, and whether this is modulated by IL1B and development. We assessed immunofluorescence both in MACS-isolated CD11B+ cells cultured from P1 mice (Fig. 4 and Supplementary Fig. 12), and in tissue sections from pups exposed to IL1B from P1 to P3 (Supplementary Fig. 13). Under control conditions at P1, microglia produce DLG4 protein that is localised to the cell membrane (>95% of IBA1+ cells stained for DLG4) (Supplementary Movie 2). This DLG4 staining disappears by P3 (Supplementary Fig. 13, postnatal day 1 and 3 PBS overlay), and the protein is still absent from IBA1-positive cells at P45. Following exposure to IL1B, DLG4 was detected at the microglial membrane at both P1 and P3 (Supplementary Fig. 13, postnatal day 1 and 3 IL1B overlay) but not P45, suggesting a delay rather than a permanent change in development, consistent with current hypotheses^42^.

### Microglia synthesise endogenous DLG4 in early development

To verify that DLG4 protein detected in microglia is endogenously synthesised, rather than the phagocytosed synaptic debris seen later in development^43^, we stained for DLG4 and lysosomal-associated membrane protein 1 (LAMP1), a marker of lysozymes. No co-localisation of these markers was demonstrable in IBA1+ cells, with LAMP1 immunofluorescence mainly confined to intracellular vesicles and DLG4 consistently and clearly at the microglial cell membrane (Supplementary Fig. 14). A 3D reconstruction of DLG4 and IBA1 immunofluorescence showed evident membranous staining (Supplementary Movie 2). In addition, in the developing cortex at P1 and P3, DLG4 protein staining was absent from cells not expressing IBA1. At P5, we observed DLG4 protein in a limited number of putative cortical neurons, in keeping with the reported normal developmental expression in the cortex in mice and comparative time points in humans^44^. We also found DLG4 protein at the membrane of MACS-isolated primary mouse microglia maintained ex vivo for 96 h, in the absence of neurons (Supplementary Fig. 12).

### DLG4 is expressed in developing and adult human brain tissue

Interrogating the BrainCloud resource^45^, we found the *DLG4* gene expressed in human cortex from early fetal development (14+ gestational weeks (GW)) throughout life, with expression increasing rapidly during the first year (Supplementary Fig. 15a)^46^. The BrainSpan Atlas of the developing human brain^47^ additionally suggests a relatively robust expression from early gestation with an apparent decrease in *DLG4* expression at around 21 GW, then increasing values from 37 GW (Supplementary Fig. 15b). The Allen Brain Atlas Brain Explorer^48^ indicates widespread cortical expression in adulthood (Supplementary Fig. 15c) and the UK Brain Expression Consortium (UKBEC) database^49^ records *DLG4* widely expressed in both grey and white matter in adults (Supplementary Fig. 8c). However, *DLG4* mRNA is targeted for degradation via specific post-translational modifications and may not be strictly predictive of protein levels^50^.

### DLG4 is expressed by microglia in the developing human brain

**Fig. 5:**
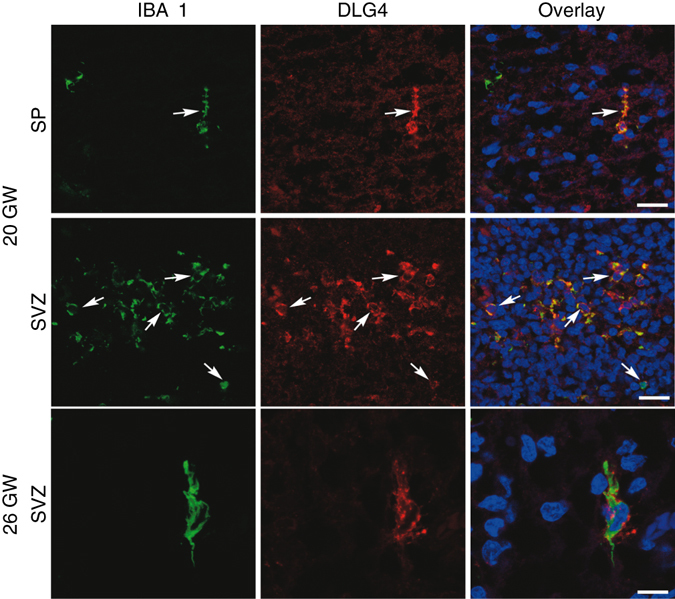
Co-localisation of DLG4 protein and microglial marker IBA1 in the developing human brain. Representative photomicrographs from the subplate (SP) and subventricular zone (SVZ) through the dorsal cortex of a 20 GW (*upper two rows*) and 26 GW (*lower row*) human brain. No IBA1 PSD95 co-localisation was observed at 30GW. In *green* IBA1+ microglia (IBA1), in *red* DLG4 (PSD95) protein and *final column* shows co-localisation of IBA1 and DLG4, together with DAPI nuclear staining. *Arrows* in *top rows* point to area of distinct co-localisation at ×20 and a higher magnification is shown of a single double-stained cell in the *lower panel*. *Scale bar* two *top rows* = 40 µm and *lower row* = 10 µm

We then assessed cell-type specific expression of DLG4 protein from microglia of the developing human brain in isolated CD11B+ cells and in tissue sections. In MACS-isolated human fetal microglia at 19 and 21 GW (roughly equivalent to P1 in mouse^15^) DLG4 protein co-localised with IBA1 (Supplementary Fig. 16); this staining appeared to increase with activation of microglia to a pro-inflammatory state using LPS. Further, we stained human fetal brain sections through the dorsal cortex. At 20 GW, there was co-localisation of IBA1 and DLG4 protein (Fig. 5) limited to cells in the proliferative zone, and the absence of any other DLG4-positive cells. At 26 GW, IBA1 and DLG4 again co-localised, but DLG4 was also detected in sparse cells, putative neurons, in the cortex. At 30 GW, we noted very few IBA1 and DLG4 co-localised cells, but many DLG4-positive cells in the cortex.

*DLG4* is thus widely expressed in the human brain during development with synthesis of DLG4 protein by human microglia at a time when there appears to be minimal expression by neurons.

### *DLG4* gene variants and matter structure in preterm infants

To assess whether the *DLG4* gene might be important in infants surviving preterm birth, we undertook an imaging-genomic analysis. We focused on white matter, which has a well-established correlation with outcome^6, 7, 51^, and a high microglia content particularly at this stage of development^2, 52^. d-MRI brain images for two separate cohorts of preterm infants (*n* = 70 and 271, Supplementary Table 9) were acquired at term-equivalent age, and a measure of white matter microstructure known to be affected by prematurity (fractional anisotropy, FA) was extracted using tract-based spatial statistics (TBSS) (Supplementary Methods). DNA extracted from saliva was genotyped on the Illumina HumanOmniExpress-12 array. Seven single nucleotide polymorphisms (SNPs) on this array mapped to *DLG4* (Supplementary Table 10), including rs17203281, which has previously been associated with neuropsychiatric disease, significantly predicting schizophrenia risk^53, 54^ and changes in cortical regional volume in patients with Williams’ syndrome, a well-characterised genetic syndrome overlapping with autism^31^.

**Fig. 6:**
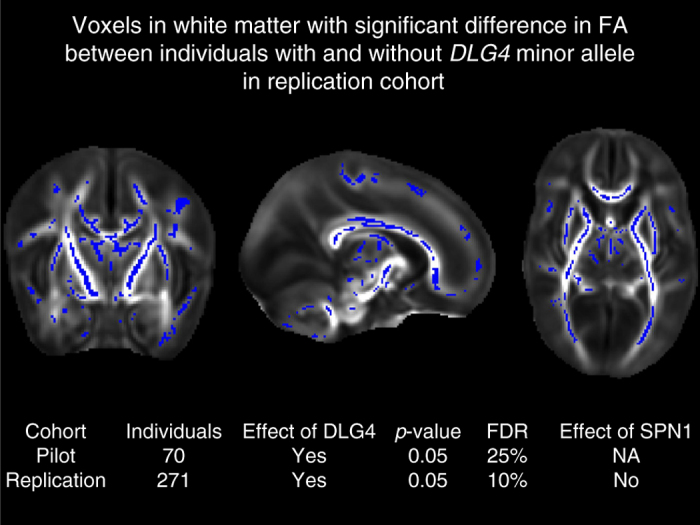
3T d-MRI brain images for two cohorts of preterm infants acquired at term-equivalent age (Pilot: cohort 1, *n* = 70; Replication: cohort 2, *n* = 271). Images from replication analysis (cohort 2) shown. Views = sagittal, coronal, axial (left-right). Coloured voxels have significantly different diffusion features (fractional anisotropy, FA) between infants with or without the minor allele for DLG4 (*n* = 271, threshold-free cluster enhancement (TFCE) *p* value <0.05, FDR=10%^81^). *Table inset*: replication of findings in two independent cohorts of preterm infants

Infants in both cohorts were categorised by presence or absence of the minor allele (A) for *DLG4* at rs17203281 to test for the effect of minor allele load (minor allele frequency (MAF) in cohort 1 (pilot) = 0.22; MAF in cohort 2 (replication) = 0.28), and a general linear model was used to test for a correlation between genotype and white matter FA. This analysis showed a significant difference (*p* < 0.05, family-wise error (FWE)-corrected by threshold-free cluster enhancement, FDR=10%) in FA between infants with or without the minor allele (A) for SNP rs17203281 in both cohorts (Fig. 6). There were no significant differences in other clinical features (gestational age at birth, age at scan, days of ventilation, incidence of chorioamnionitis, bacterial sepsis or necrotising enterocolitis, Supplementary Table 15) between infants with or without the minor allele. None of the other SNPs at the *DLG4* locus had a replicable effect in both cohorts.

To investigate whether the observed effect of common genetic variation in *DLG4* is specific to this gene or could be extended to SPN1 genetic variants as a group, we used a list of all genes in SPN1 as a set of interest and carried out association testing with the phenotype using joint association of genetic variants^55^ in the larger cohort of 271 infants. This tests the null hypothesis of there being no more evidence for association of common genetic variants (SNPs) in genes from SPN1 with the phenotype under study than any other random set of an equal effective number of SNPs (see “Methods” section). The SPN1-gene set was tested for association with the real phenotype (query data), and this was repeated with 1000 permutations of the phenotype (self-contained test) to estimate empirical *p* values. This analysis showed no significant effect of the genes in SPN1 on FA, supporting the specific association of *DLG4* with white matter features in the developing brain rather than a wider and more general effect of the larger SPN1 network.

### eQTL effect of *DLG4* in human brain

We then queried whether rs17203281 in the *DLG4* region might be regulating *DLG4* mRNA expression, and searched for a possible expression quantitative trait locus (eQTL) effect. We analysed *DLG4* mRNA expression within individual tissues in the brain, treating the expression levels of the gene as a quantitative trait, so that variations in gene expression that are highly correlated with genetic variation can be identified as eQTLs. A SNP located within or in close proximity to a gene that is significantly associated with the gene’s mRNA variation defines a *cis*-acting eQTL. We returned to the UKBEC database and extracted genotypes for the rs17203281 plus *DLG4* gene expression in the white matter, and compared gene expression levels to genotypes. This analysis suggested that normal individuals homozygote for the rs17203281 minor allele (AA) genotype have a significantly higher expression of *DLG4* in white matter than those with the alternate genotypes (AG or GG) (Student’s *t* test, *p* < 0.05), suggesting a *cis*-eQTL effect for rs17203281 (Supplementary Fig. 17).

To cross-validate this result, we queried the GTEx portal, which provides a searchable resource of multiple different human tissues with genotyping, gene expression profiling, whole-genome sequencing and RNA sequencing data^56^. This revealed that *DLG4* is preferentially expressed in the brain (Supplementary Fig. 8b), and has multiple SNPs regulating its expression levels in *cis* (Supplementary Table 11), of which one SNP (rs3826408) is in high linkage disequilibrium (i.e., is a proxy SNP) with rs17203281 (*r* ^2^ = 0.667, *D*′ = 1). Together, these analyses suggest *cis*-acting genetic regulation of *DLG4* mRNA expression in the brain by rs17203281.

### Overlaps between SPNs and neuropsychiatric gene modules

Rare genetic variants in *DLG4* have previously been associated with autism, schizophrenia and epilepsy^30^. This identified *DLG4* within a small module of 24 genes involved with synaptic function, in which de novo and more severe missense mutations were more likely in individuals with significantly higher intellectual impairment. We investigated the overlaps between the main gene modules reported in ref. ^30^ and genes in SPN1 and SPN2 using a formal test for intersecting gene lists^57^ (Supplementary Fig. 18 and Supplementary Table 12). We found that there were significant overlaps between SPN1/2 and the reported disease-associated modules. In particular, *DLG4* was the most frequently occurring gene in these overlaps (11/51 occurrences) followed by another gene from SPN1, *CAMK2A* (4/51). Notably, SPN1/2 were broadly captured by the autism modules 1/2 reported in ref. ^30^.

## Discussion

The primary finding of this study is that DLG4 is expressed in microglia during early development in a developmentally regulated expression pattern that is altered by neuroinflammation associated with brain damage in preterm born infants. This is a novel role for DLG4, previously considered an archetypal marker of the neuronal post-synaptic density.

The IL1B administration model used here captures essential features of the encephalopathy of prematurity including hypomyelination linked to oligodendrocyte maturation arrest, microglial activation, cognitive deficits, decreased fractional anisotropy on MRI and axonopathy^14^. Systemic IL1B generated two waves of gene expression in microglia, with upregulation of inflammatory genes and downregulation of genes involved in growth, creating a genomic storm^20^ that altered the normal gene and protein network topology. DLG4 was the hub of the SPN1 protein network that is highly enriched with genes implicated in neurocognitive disorders. SPN1 has a star configuration, which appears to maximise network efficiency at the expense of robustness, since perturbing the central hub broadly disrupts the system^58^. Hub proteins are important for cellular growth, under tight regulation, continuously evolving^59^, and more intrinsically disordered than proteins with fewer relations^60^. DLG4 is the only member of either SPN1 or SPN2 that qualifies as a hub, and is significantly enriched for disorder promoting amino acids (Student’s *t* test, *p* < 0.02)^61^.

SPN1 interacted with SPN2 via the transcriptional regulation by STAT3, a TF previously linked to microglial activation and neuroprotection pathways^62^. The combined PPI and TF analysis revealed a possible functional relationship between two protein interaction sub-networks. The same relationship was not observed between the STATs TFs and genes in SPN2.

Microglia DLG4 protein synthesis at P1 disappears by P3, unless this developmental pattern is disrupted by inflammation leading to the temporary persistence of DLG4 at the cell membrane, which resolves by P45. The disappearance of DLG4 protein between P1 and P3 could be due to post-transcriptional mechanisms involving micro-RNAs, mRNA degradation and post-translational mechanisms affecting protein localisation as previously observed in neurons (review in ref. ^50^). Inflammation-induced persistence of DLG4 protein may be a previously unappreciated mechanism of inflammatory brain injury.

In human infants, we found developmentally regulated *DLG4* gene expression throughout brain tissue. In mouse and human microglia, DLG4 protein localised to cell membranes and was not found within cytosolic vesicles: DLG4 detection was thus not due to the participation of microglia in synaptic pruning seen later in development^43^, reflecting the well-known role of DLG4 in synapse structure and development^32, 41, 46^; indeed this is consistent with previous data showing that *DLG4* mRNA was not expressed in neurons before P5-10^44^.

Common genetic variation in *DLG4* (rs17203281) was associated with structural white matter changes in two independent infant cohorts, possibly due to measured differences in expression of *DLG4* mediated through a posited *cis*-eQTL action of rs17203281, suggesting that inter-individual genetic variability in *DLG4* gene could affect the response to perinatal inflammation. Preterm infants have an increased risk of developing autism spectrum disorders (ASD) and other neuropsychiatric disorders, and *DLG4* has been consistently associated with neuropsychiatric diseases including ASD and schizophrenia^30, 63^ (Supplementary Data 11), while mice with *Dlg4* deletion (*Dlg4* ^−/−^) exhibit increased repetitive behaviour, abnormal communication and social behaviour^31^.

The observed changes in d-MRI are characteristic of encephalopathy of prematurity. However, d-MRI provides no information on the cell types involved in these microstructural abnormalities, and the relationship between *DLG4* variability and brain structure cannot be attributed specifically to microglia. Nevertheless, white matter is highly populated with microglia in the perinatal period in both humans and mouse^52^, and even in adults microglia are typically much more numerous in white matter than in neocortex^64^. TBSS provides a powerful approach to d-MRI data, providing group analyses that overcome problems of partial volume effects. However, TBSS is highly dependent on the quality of the image registrations^65, 66^, and as this is particularly challenging in developing brain, we have created an optimised neonatal pipeline and characterised the sensitivity of TBSS in this population^67, 68^. In addition, although TBSS is a powerful technique for identifying associations with white matter microstructure, it is less reliable for precise spatial localisation within white matter.

The mechanism of DLG4 action in microglia remains unclear. In vitro, inhibition of the interaction between DLG4 protein and the *N*-methyl-D-aspartate receptor N2B subunit^69^ (which has a moderate but significant effect on microglial activation state and response to perinatal injury^70^) had no effect on microglia phenotype. We conjecture that the role of DLG4 in microglia may be in the domain of cell-cell communications, such as cross-talk between oligodendrocytes, astrocytes and microglia^71^ or regulation of glutamatergic and GABAergic signalling^72^. Of interest, DLG4 may be involved with the clustering and activity of inwardly rectifying potassium channels (Kir) predominantly expressed in glial cells^73^. Microglial Kir have functional effects on microglial activity and could have therapeutic applications in Alzheimer’s and Parkinson’s diseases^74^. DLG4 has also been observed in oligodendrocytes and may mediate Kir-related myelination^75^.

The inflammatory reflex involving the vagus nerve^76^ could attenuate the IL1B response in vivo, and microglia activity may be modulated via microglial α7 nicotinic acetylcholine receptors (α7AChRs) that limit microglial activation^77^, possibly signalling via NRG/ErbB4 interactions with DLG4 in microglia^78^ or via endothelial COX2^79^. This might be a fruitful area for further discovery. It is also unclear how much the microglial response is conditioned by cellular development and context, and whether this might differ between the in vivo and in vitro settings, as well as between mouse and human. The topic of microglial development including how to best characterise it in relation to function requires further work.

## Methods

### Animal model

Experimental protocols were approved by the institutional guidelines of the Institut National de la Santé et de la Recherche Scientifique (Inserm, France), and met the guidelines for the United States Public Health Service’s Policy on Humane Care and Use of Laboratory Animals (NIH, Bethesda, MD, USA). The experimental set-up for inducing inflammation-induced white matter injury in the mouse has previously been described in detail^14^. In brief, a 5 μl volume of phosphate-buffered saline (PBS) containing 10 μg/kg injection of recombinant mouse IL1B or of PBS alone (control) was injected intra-peritoneally twice a day (morning and evening) on days postnatal P1-P4 and once in the morning on day P5. Animals were sacrificed 4 h after the morning injection of IL1B at P1, P5, P10 and P45. Brains from animals at P1, P3 and P10 were also used for immunofluorescence with antibodies to detect DLG4, ((6G6-1C9) (Product# MA1-045), Thermo Scientific; 1:500), ((IBA1) (ab5076, Abcam; 1:400) and LAMP1 (L1418, Sigma; 1:200). Details in Supplementary Methods.

### CD11B+ microglia MACS in mouse

Brains were collected from mice for cell dissociation, and microglia were isolated by magnetic antibody-based cell sorting (MACS) using CD11B antibody (Miltenyi Biotec, Bergisch Gladbach, Germany, dilution 1:10) (details in Supplementary Methods) according to the manufacturer’s protocol using all recommended reagents and equipment. The purity of MACSed CD11B+ fractions was validated using FACS analysis of CD11B fluorescence, and the purity was further validated with RT-qPCR of the positive and negative CD11B cell fractions. Details in Supplementary Methods.

### Gene co-expression network reconstruction

Mouse microglial RNA was extracted and hybridised to Agilent Whole Mouse Genome Oligo Microarrays (8 × 60 K). Three separate gene co-expression networks were reconstructed from these data, showing a significant response to IL1B, Development and Interaction effect (Fig. 2). Details in Supplementary Methods.

### PPIs and transcriptional regulation

The nodes of all three gene networks (IL1B, Development and Interaction) were aggregated into one list and used to investigate protein interactions with a PGA^23^, and two super-power nodes (SPNs) were identified by examining which sets of power nodes were directly interconnected (Fig. 3 and Supplementary Fig. 6). GO annotation and enrichment analysis was carried out, and the transcriptional control of the protein networks was interrogated using motif analysis and ChIP-Chip data^37^. Details in Supplementary Methods.

### Mouse cell culture studies

MACS-isolated microglia were stimulated by IL1B + IFNg and exposed to one of: vehicle; a small molecule inhibitor of STAT3 (BP-1-102; Merck Millipore) or TAT-N-dimer (an inhibitor of the DLG4 protein NMDA receptor interaction; Merck Millipore). At the end of the treatment period, cells were harvested and used for immunofluorescent labelling; mRNA was extracted for gene expression analysis, and supernatant was collected for nitrites/nitrates or cytokines/chemokines measurement. Antibodies used were a mouse monoclonal antibody to detect DLG4 ((6G6-1C9) (Product# MA1-045), Thermo Scientific; 1:500), a goat polyclonal antibody to detect IBA1 Ionized calcium binding adaptor molecule 1 ((IBA1) (ab5076, Abcam; 1:400)), and a rabbit polyclonal antibody to detect Lysosomal-associated membrane protein 1 ((LAMP-1) (L1418, Sigma; 1:200)). Details in Supplementary Methods.

### Developing human brain post-mortem studies

All human post-mortem tissue (cells and tissues) was acquired with ethical approval at The French Agency of Biomedicine (Agence de Biomédicine; approval PFS12-0011). Written informed consent was received prior to donation of foetal tissue. For the collection of human microglia, post-mortem tissue without any neuropathological alterations was acquired from two individuals at 19 and 21 GW. MACS-isolated microglia were obtained and used for treatment with DMEM (control) or LPS, as well as immunofluorescence analysis and RT-qPCR. Human brain sections were obtained from post-mortem cases from medical abortions at 20, 26 and 30 GW for non-neurological diagnoses, and stained for IBA1 (ab5076, Abcam; 1:400) and DLG4 ((6G6-1C9) (Product# MA1-045), Thermo Scientific; 1:500). Details in Supplementary Methods.

### Infant MRI studies

Research was carried out in compliance with the Code of Ethics of the World Medical Association (Declaration of Helsinki), with approval from the NHS National Research Ethics Service (NRES), to the standard of the associated granting agencies. Two independent cohorts of preterm infants (cohort 1: *n* = 70; cohort 2: *n* = 271) were imaged at term-equivalent age on a Philips 3-Tesla system (Philips Medical Systems, the Netherlands), with acquisition of structural (T2-weighted fast-spin echo MRI and 3D-MPRAGE) and diffusion (single-shot echo-planar diffusion tensor imaging (EPI DTI)) sequences. Details in Supplementary Methods.

### Infant genotyping

Saliva samples for both infant cohorts above were genotyped on the Illumina HumanOmniExpress-12 array, as previously described^80^ (Supplementary Methods). The genotype matrix was recoded in terms of minor allele counts, including only SNPs with MAF ≥5% and ≥ 99% genotyping rate. Details in Supplementary Methods.

### Data availability

All new data are available from the authors on request, subject to ethical restrictions. Expression profiling of human dorsolateral prefrontal data is accessible in the NCBI GEO database under accession code GSE30272.

## Electronic supplementary material

Supplementary Information

Supplementary Data 1

Supplementary Data 2

Supplementary Data 3

Supplementary Data 4

Supplementary Data 5

Supplementary Data 6

Supplementary Data 7

Supplementary Data 8

Supplementary Data 9

Supplementary Data 10

Supplementary Data 11

Supplementary Movie 1

Supplementary Movie 2
