# Supplementary material for: Integrative genomics of microglia implicates DLG4 (PSD95) in the white matter development of preterm infants
Source: Nat Commun. 2017 Sep 5;8:428. doi: 10.1038/s41467-017-00422-w (PMC5585205; doi:10.1038/s41467-017-00422-w)
Supplement: Supplementary file 1 — Supplementary Information [file 41467_2017_422_MOESM1_ESM.pdf]

File name: Supplementary Information
Description: Supplementary figures, supplementary tables, supplementary methods and supplementary references.

File name: Supplementary Data 1
Description: Lists of differentially expressed genes found by MANOVA testing for each of the three responses (IL1B, Development, Interaction). High coefficient of variation (>50th centile) and correction for multiple testing (p-value < 0.05 (FDR 10%)).

File name: Supplementary Data 2
Description: KEGG annotation of differentially expressed genes found by MANOVA testing for each of the three responses (IL1B, Development, Interaction), indicating significantly over-represented biological pathways.

File name: Supplementary Data 3
Description: Gene Ontology (GO) annotation of differentially expressed genes found by MANOVA testing for each of the three responses (IL1B, Development, Interaction), indicating significantly over-represented GO terms.

File name: Supplementary Data 4
Description: Summary of representative Gene Ontology (GO) terms for the aggregated differentially expressed gene lists found by MANOVA testing for each of the three responses (IL1B, Development and Interaction), using the REVIGO tool. Frequency is the percentage of human proteins in UniProt that were annotated with a GO term in the GOA database, i.e. a higher frequency denotes a more general term. Uniqueness measures whether the term is an outlier when compared semantically to the whole list, which were calculated as 1-(average semantic similarity of a term to all other terms). More unique terms tend to be less dispensable. Dispensability is the semantic similarity threshold at which the term was removed from the list and assigned to a cluster. Cluster representatives always have dispensability below the cutoff threshold.

File name: Supplementary Data 5
Description: Full annotation of clusters of genes with patterns of response to IL1B (Figure 2b), identified when gene expression profiles were clustered by their response to IL1B at each time-point compared to controls (p<0.05, False Discovery Rate (FDR) = 10%).

File name: Supplementary Data 6
Description: Representative Gene Ontology (GO) terms for each of the three gene networks (IL1B, Development and Interaction), using the REVIGO tool as before.

File name: Supplementary Data 7
Description: Gene expression collections containing significant up-regulation of SPN1 genes (GTEx portal), identified by the Enrichr tool. Combined score: p-value computed using the Fisher exact test combined with the z-score of the deviation from the expected rank: $c = \log(p).z$

File name: Supplementary Data 8
Description: Gene expression collections containing significant up-regulation of SPN2 genes (GTEx portal), identified with the Enrichr tool, as for SPN1.

File name: Supplementary Data 9
Description: Disease term enrichment for super-power nodes (SPN1 and SPN2), within the Psychology and Psychiatry category and for Systemic Diseases in SPN1 (GDA tool, Park et. al, 2014).

File name: Supplementary Data 10
Description: Disease term enrichment for super-power node 2 for Systemic Diseases (GDA tool, Park et. al, 2014). MeSH taxonomy used for pooling genes within diseases, significance assessed through permutation (10,000 permutations).

File name: Supplementary Data 11
Description: Gene-disease evidence for DLG4 from several public data sources and the literature (DisGeNET, Piñero et al, 2015). DisGeNET gene-disease score takes into account the number and type of sources (level of curation, organisms), and the number of publications supporting the association. The score ranges from 0 to 1 (1 = highest confidence).

File name: Supplementary Movie 1
Description: Hiveplots of gene co-expression networks. Animation of progression of hiveplots through different axis ranges within the parameters in Supplementary Table 14. As the degree range allocated to each axis changes, nodes (genes) migrate to different axes depending on their degree.

File name: Supplementary Movie 2
Description: DLG4 localization in microglial cells. 3D reconstruction of a microglial cell expressing IBA1 (green) and DLG4 (red) in the cortex of a P1 animal exposed to IL1B. Note that DLG4 immunoreactivity is predominantly located at the surface of the cell body and ramifications. Frame: 30x30µm.

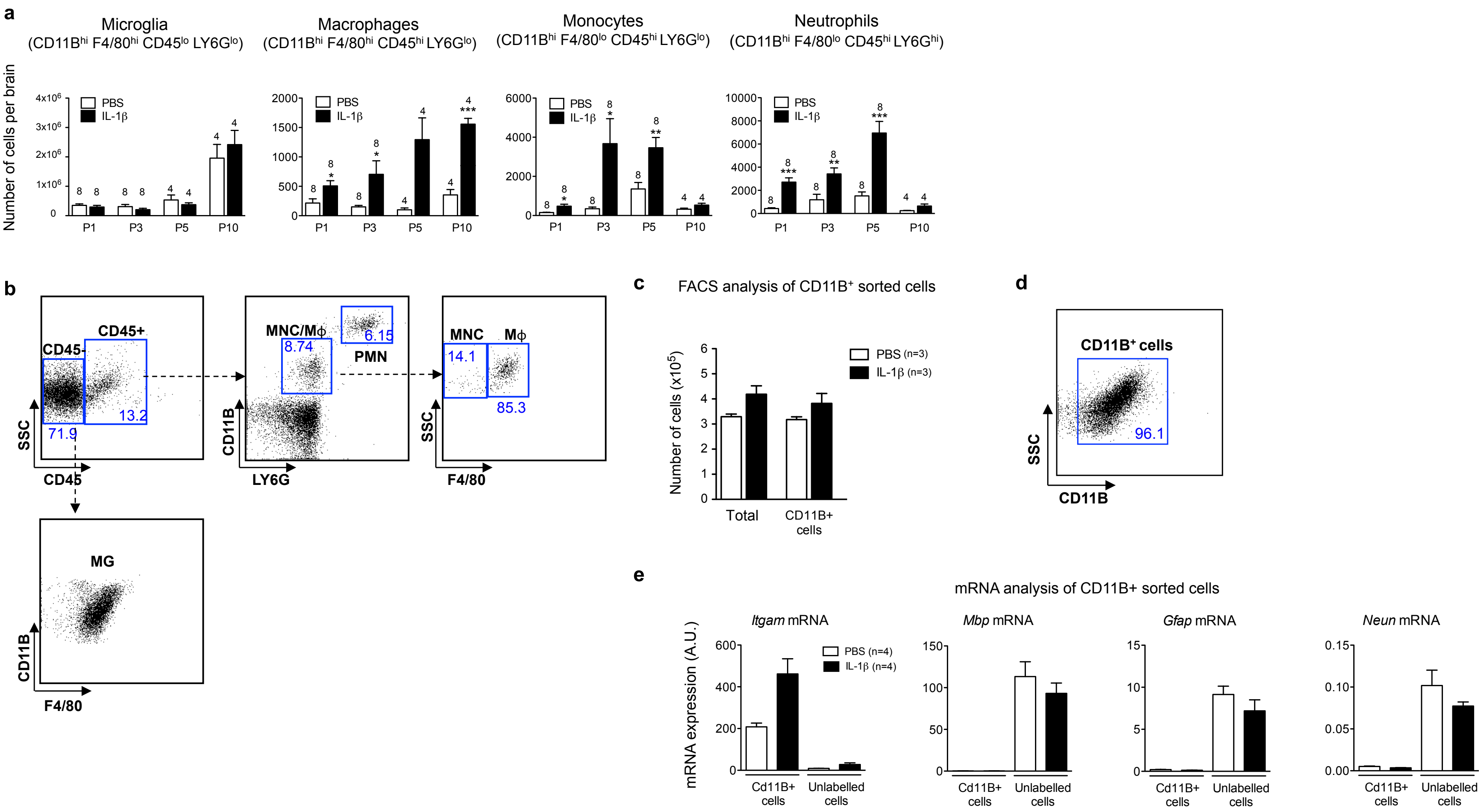
a
Microglia
(CD11Bhi F4/80hi CD45lo LY6Glo)
Macrophages
(CD11Bhi F4/80hi CD45hi LY6Glo)
Monocytes
(CD11Bhi F4/80lo CD45hi LY6Glo)
Neutrophils
(CD11Bhi F4/80lo CD45hi LY6Ghi)
Number of cells per brain
PBS
IL-1β
P1
P3
P5
P10
b
CD45-
CD45+
71.9
13.2
SSC
CD45
MNC/Mϕ
8.74
6.15
PMN
CD11B
LY6G
MNC
Mϕ
14.1
85.3
SSC
F4/80
MG
CD11B
F4/80
c
FACS analysis of CD11B+ sorted cells
Number of cells (x10^5)
PBS (n=3)
IL-1β (n=3)
Total
CD11B+ cells
d
CD11B+ cells
96.1
SSC
CD11B
e
mRNA analysis of CD11B+ sorted cells
Itgam mRNA
Mbp mRNA
Gfap mRNA
Neun mRNA
mRNA expression (A.U.)
PBS (n=4)
IL-1β (n=4)
Cd11B+ cells
Unlabelled cells

Supplementary Figure 1 Characterisation of CD11B positive cells and validation of MACS CD11B positive cell isolation purity. Myeloid cells in brain of mice injected with IL1B, and analysis of magnetically sorted (MACS® technology) Cd11b+ cells from P1 brain of PBS or IL1B treated mice. (a) Quantification of the number of myeloid cells (MG, macrophages, monocytes and neutrophils) in whole brain at P1, 3, 5 and 10 from PBS or IL1B treated mice by FACS analysis. (n=4-9/group, mean ± SEM), * $p<0.05$, ** $p<0.01$ and *** $p<0.001$; Mann and Whitney's test. (b) Representative flow cytometry dot plot for myeloid populations in brain of mice. MG (MG) are defined as CD11bhi CD45lo cells, Neutrophils (PMN) defined as CD11bhi CD45hi Ly6Ghi, Monocytes (MNC) defined as CD11bhi CD45hi Ly6Glo F4/80lo cells and macrophages (MP) defined as CD11bhi CD45hi Ly6Glo F4/80hi cells. (c) Analysis of magnetically sorted (MACS® technology) Cd11B+ cells isolated from P1 brain of PBS or IL1B treated mice by FACS analysis. (n=3/group, mean ± SEM). More than 96% of cells were CD11Bhi. (d) Representative flow cytometry dot plot for sorted (MACS® technology) CD11B+ cells from P1 brain of PBS or IL1B treated mice. (e) RT-qPCR analysis of Itgam, Mbp, Gfap and Neun mRNA expression on MACSed brain Cd11b+ cells and unlabeled cells. mRNA levels are presented as a fold change relative control after normalization with the housekeeping gene (Rpl13a). All quantitative data of this figure are expressed as the mean ± SEM, (n=4/group).

a

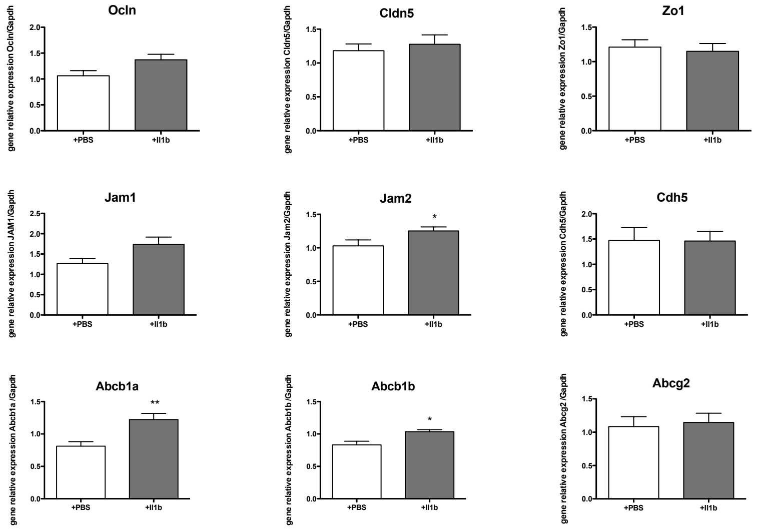

b

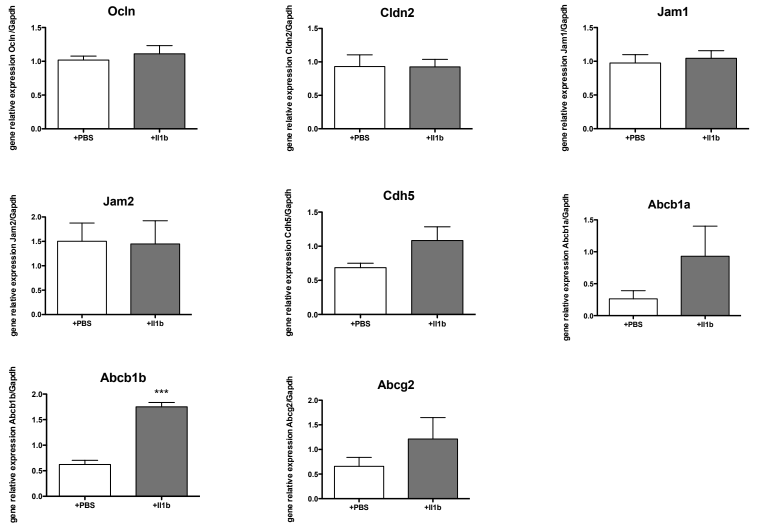

c

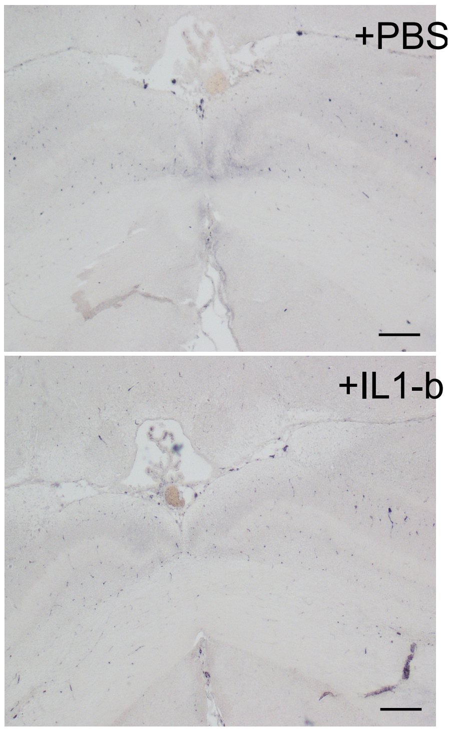

d

Gene	Sequence
Abcb1a	CATACAAATGCCATCCATGC<br>CCCCTTCCTTTTCCTTGCTA
Abcb1b	AGAACGCAGACTTGATCGTG<br>CAGCCTGAACCATCGAGAA
Abcg2	ACCCACAGGCGGAGGCAAGT<br>GCAGGTTGAGGTGCCCCGTTT
Cdh5	CGC CAA AAG AGA GAC TGG AT<br>TTC TGA CGG TTC ACA TTG GA
Cldn2	GCTCCGTGAGTATCTGCTCTG<br>TCACAGTGTCTCTGGCAAGC
Cldn5	TGA GCA TTC GGT CTT TAG CC<br>ACC CAG CCT ACC AGA CAC AG
JAM1	CCGTGCCTTCATCAATTCTT<br>ATGCGTACAGCCTCTGACCT
JAM2	TGT GGA GCT ACG GTG TCA AG<br>TAC GAG CTG TTC CTG TGT GC
Ocln	ATG AAC AGC CCC CTA ATG TG<br>CTT TGC CGT TGG AGG AGT AG
ZO1	GTCGCAATGGTTAACGGAGT<br>CGTGCACATCCTCGTCATAG

Supplementary Figure 2 Blood choroid plexus barrier data.

Analysis of gene expression levels (RT-qPCR) of brain barrier components. Tight junctions (Ocln, Cldn5, Zo1, Jam1, Jam2, Cldn2), adherens junctions (Cdh5), transporters (Abcb1a, Abcb1b, Abcg2) in the cortex blood brain barrier, (A) and in the plexus blood-CSF barrier, (B) of P5 rats with intraperitoneal injections of PBS or IL1B. Low-magnification images of IgG immunolabeling in the brain of P5 rats with intraperitoneal injections of PBS or IL1B (C) scale bar: 200µm. (animals were sacrificed 3h after last injection, 8 animals per group).

Complete Gene Ontology annotation for differentially expressed genes

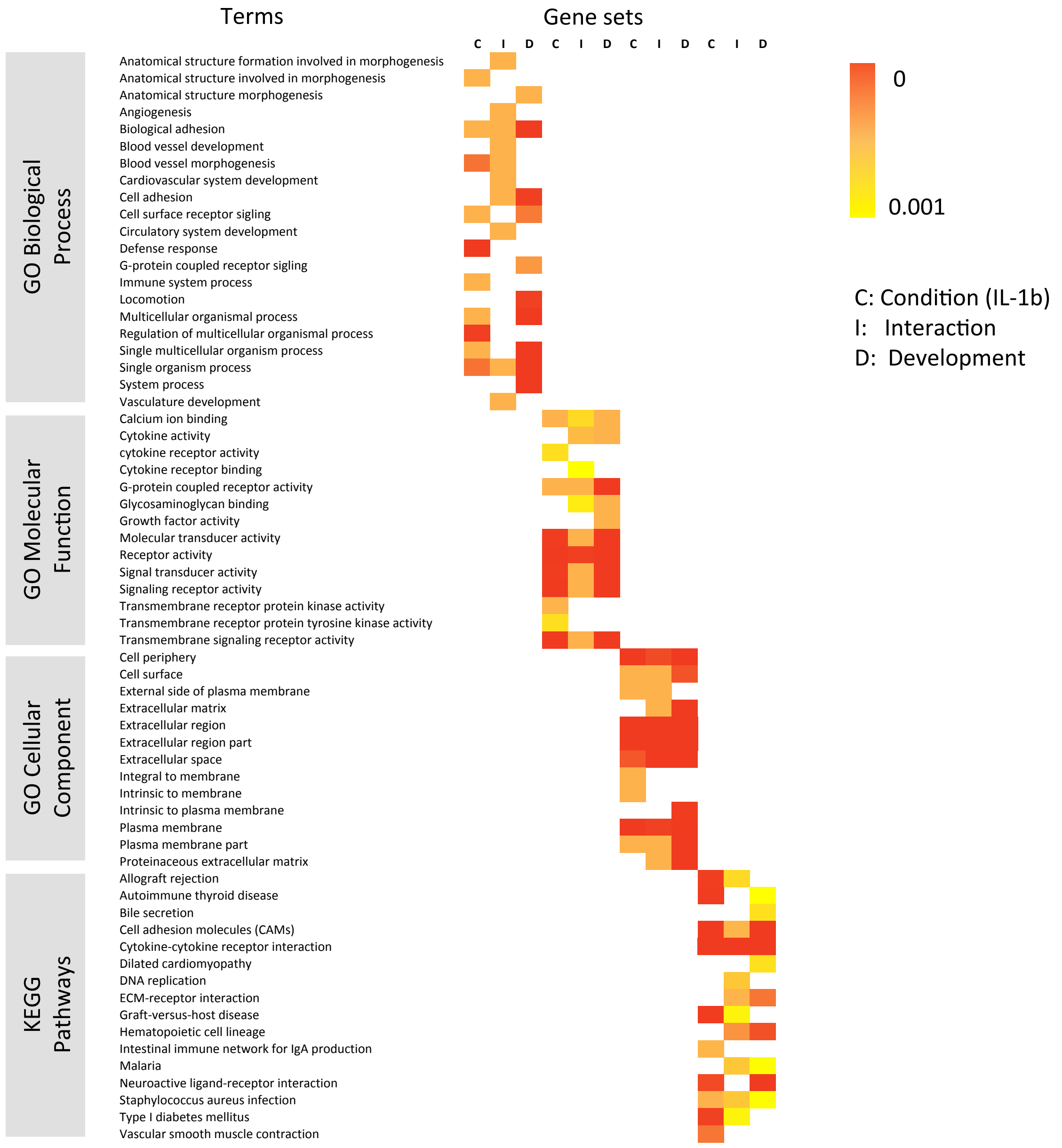

Supplementary Figure 3 Complete Gene Ontology enrichment annotation for differentially expressed genes, by response.
Responses: Condition (i.e. IL1B exposure), Development, Interaction), adjp < 0.01 (correction with Benjamini & Hochberg (1995), see Methods).

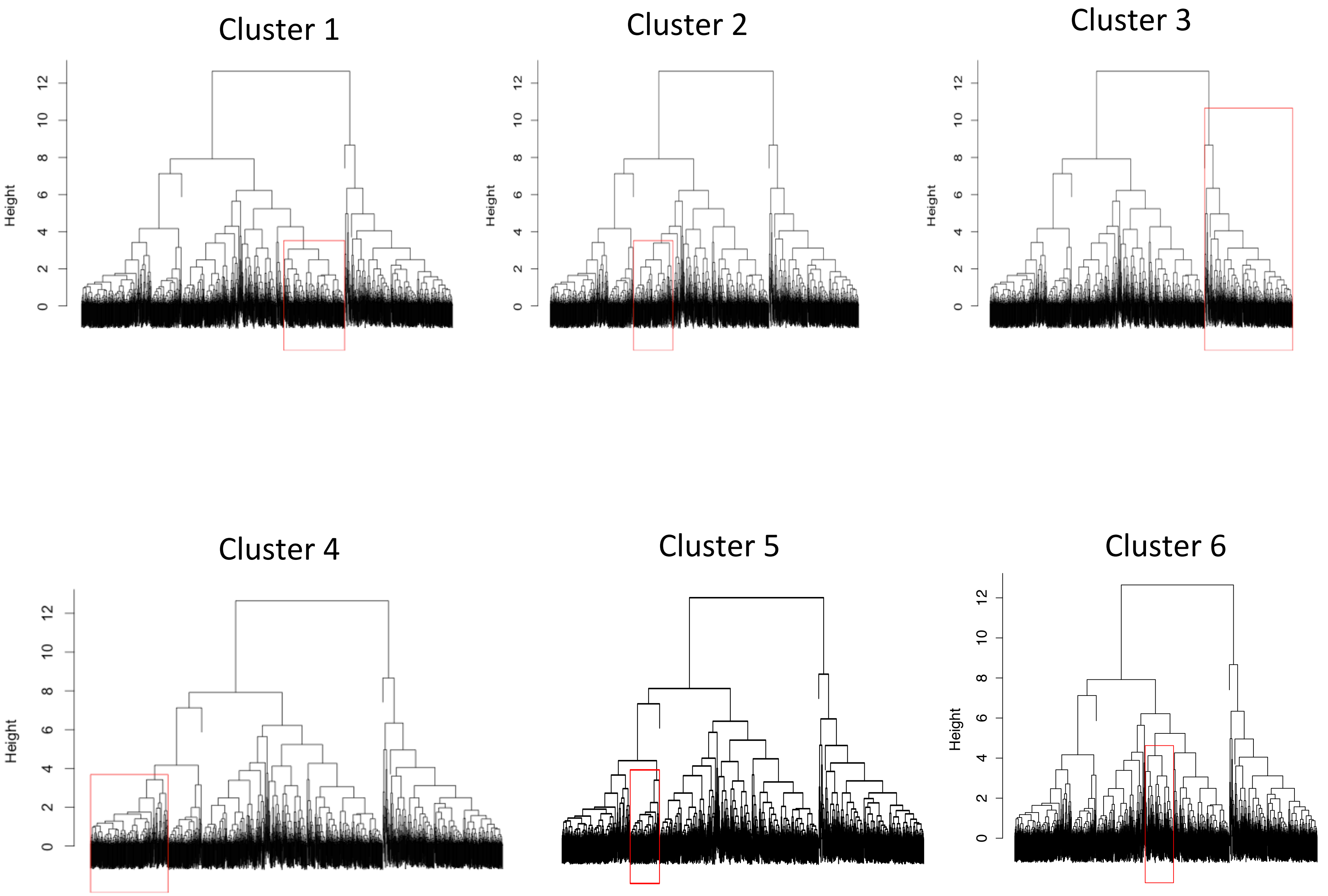
Cluster 1
Cluster 2
Cluster 3
Cluster 4
Cluster 5
Cluster 6
Height
0
2
4
6
8
10
12

Supplementary Figure 4 Clusters of genes by expression profile similarity in response to IL1B. Please refer also to Figure 1, panel b.

Complete Gene Ontology annotation for gene networks

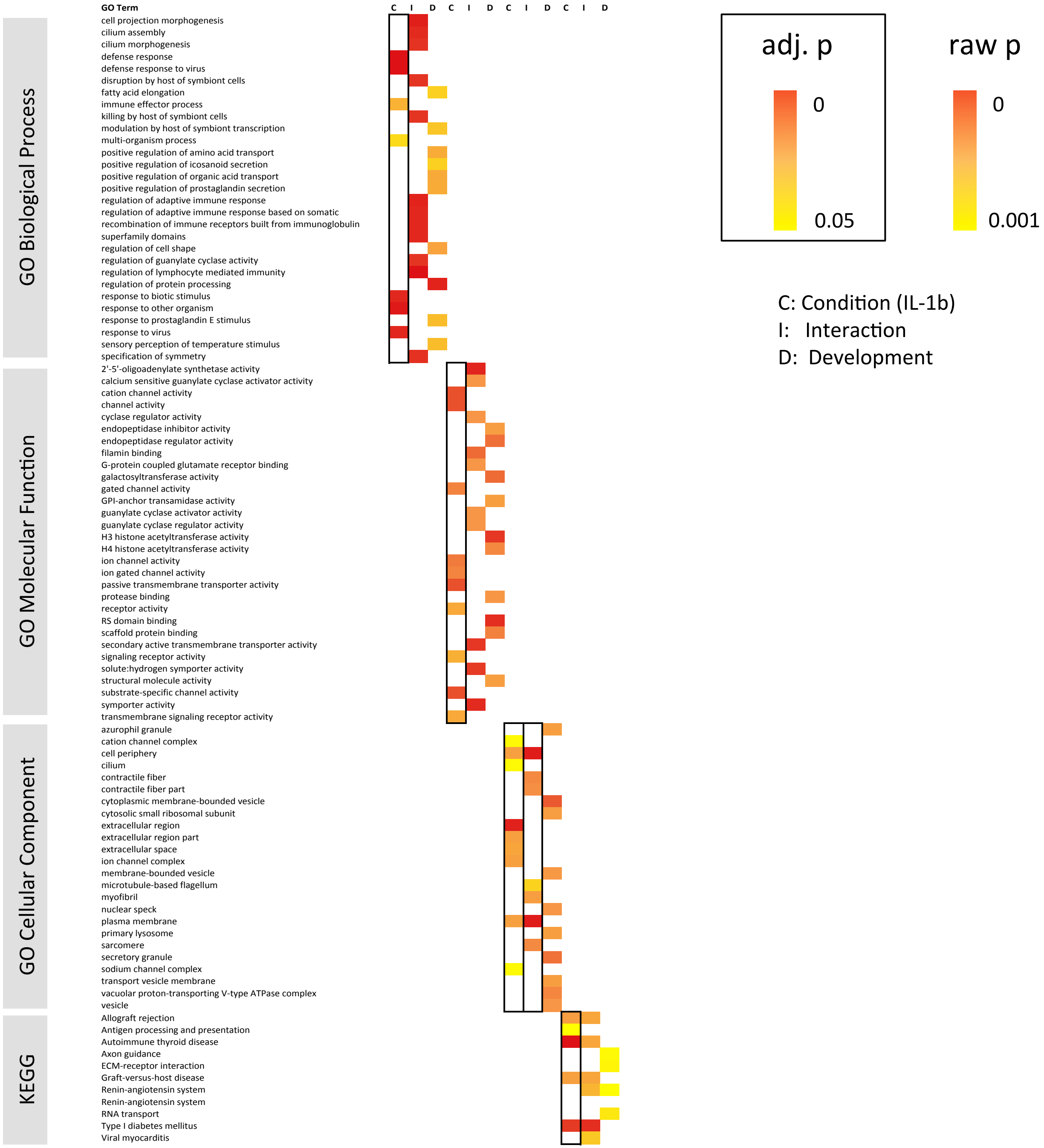

Supplementary Figure 5 Complete Gene Ontology enrichment annotation for genes in each co- expression network. Condition (i.e. IL1B exposure), Development, Interaction). Annotations with adjusted $p < 0.05$ (correction with Benjamini & Hochberg (1995), see Methods) are outlined in black boxes.

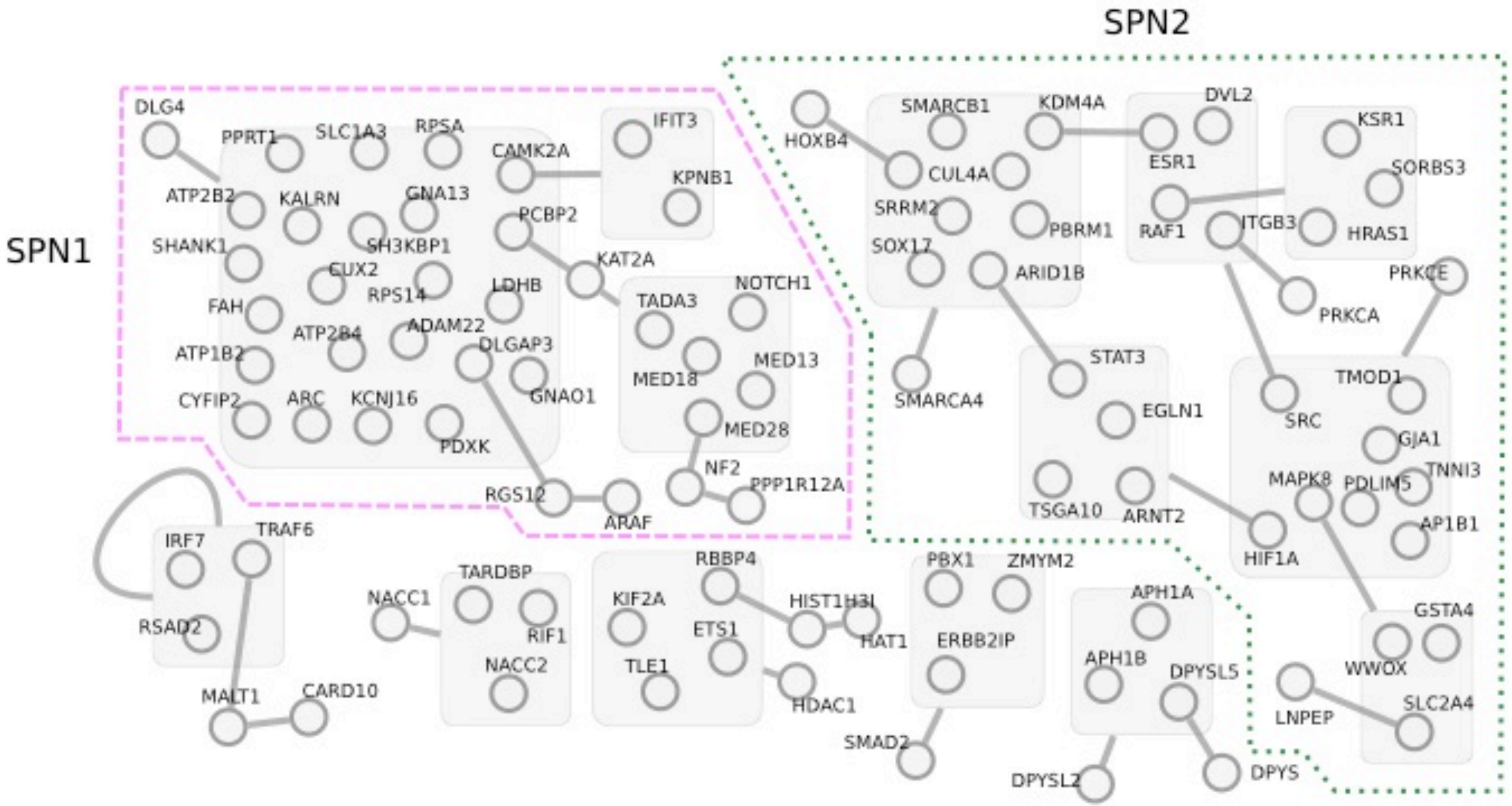
SPN1
SPN2
DLG4
PPRT1
SLC1A3
RPSA
CAMK2A
IFIT3
KPNB1
ATP2B2
KALRN
GNA13
PCBP2
SHANK1
SH3KBP1
CUX2
KAT2A
RPS14
LDHB
FAH
ATP2B4
ADAM22
DLGAP3
ATP1B2
GNAO1
CYFIP2
ARC
KCNJ16
PDXK
TADA3
NOTCH1
MED18
MED13
MED28
NF2
PPP1R12A
RGS12
ARAF
IRF7
TRAF6
RSAD2
MALT1
CARD10
NACC1
TARDBP
RIF1
NACC2
KIF2A
RBBP4
ETS1
TLE1
HIST1H3I
HAT1
HDAC1
HOXB4
SMARCB1
KDM4A
CUL4A
SRRM2
PBRM1
SOX17
ARID1B
SMARCA4
DVL2
ESR1
RAF1
ITGB3
KSR1
SORBS3
HRAS1
PRKCE
PRKCA
STAT3
EGLN1
TSGA10
ARNT2
TMOD1
SRC
GJA1
TNNI3
MAPK8
PDLIM5
AP1B1
HIF1A
PBX1
ZMYM2
ERBB2IP
SMAD2
APH1A
APH1B
DPYSL5
DPYSL2
DPYS
GSTA4
WWOX
LNPEP
SLC2A4

Supplementary Figure 6 Complete power graph analysis (PGA) plot.
Two super-power nodes (SPNs) contained in dashed outlines and used in subsequent analysis.

Significance of Dlg4 and Stat3 subnetworks (Netvenn vs Dapple)

SPN	PARAMETER	OBSERVED IN DAPPLE	EXPECTED	P-VAL	OBSERVED IN NETVENN
SPN1	Direct Edge Count	23	4.911	<0.001	36
SPN2	Direct Edge Count	36	9.68	<0.001	41

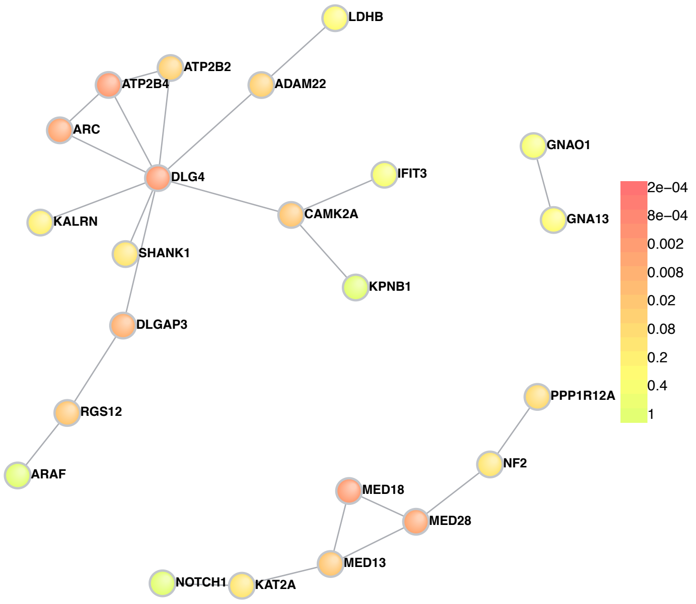

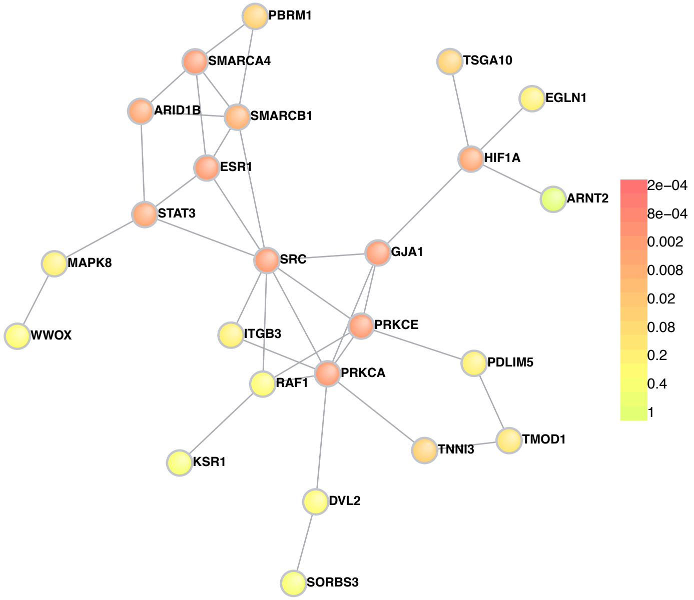

Supplementary Figure 7 Protein-protein interactions (PPI) reconstructed by DAPPLE (Broad Institute). Network p-values based on 1000 permutations, and comparison to PPI from power-graph analysis (Netvenn).

a

Glia-specific gene interactions

SPN1 SPN2

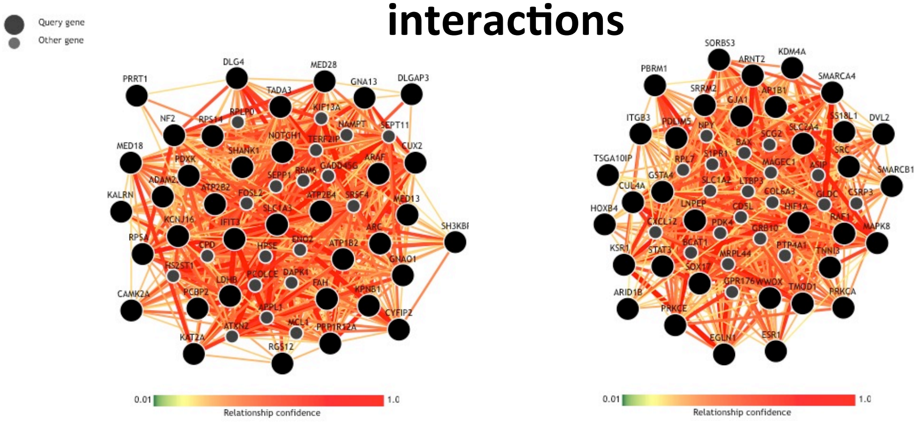

c

DLG4 brain expression by region

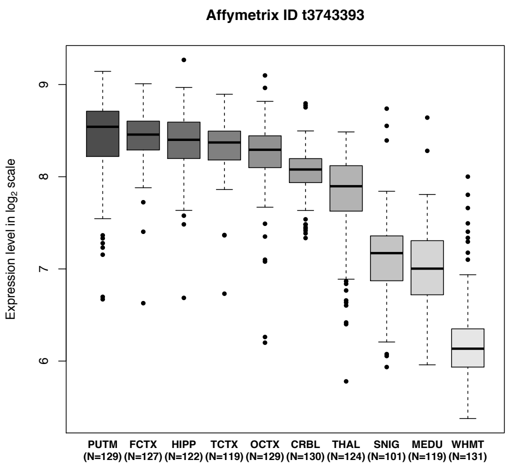

Abbreviation	Region
PUTM	Putamen
FCTX	Frontal cortex
HIPP	Hippocampus
TCTX	Temporal cortex
OCTX	Occipital cortex
CRBL	Cerebellar cortex
THAL	Thalamus
SNIG	Substantia nigra
MEDU	Medulla (inf. olivary nucleus)
**WHMT**	**Intralobular white matter**

b

DLG4 expression across tissues in humans

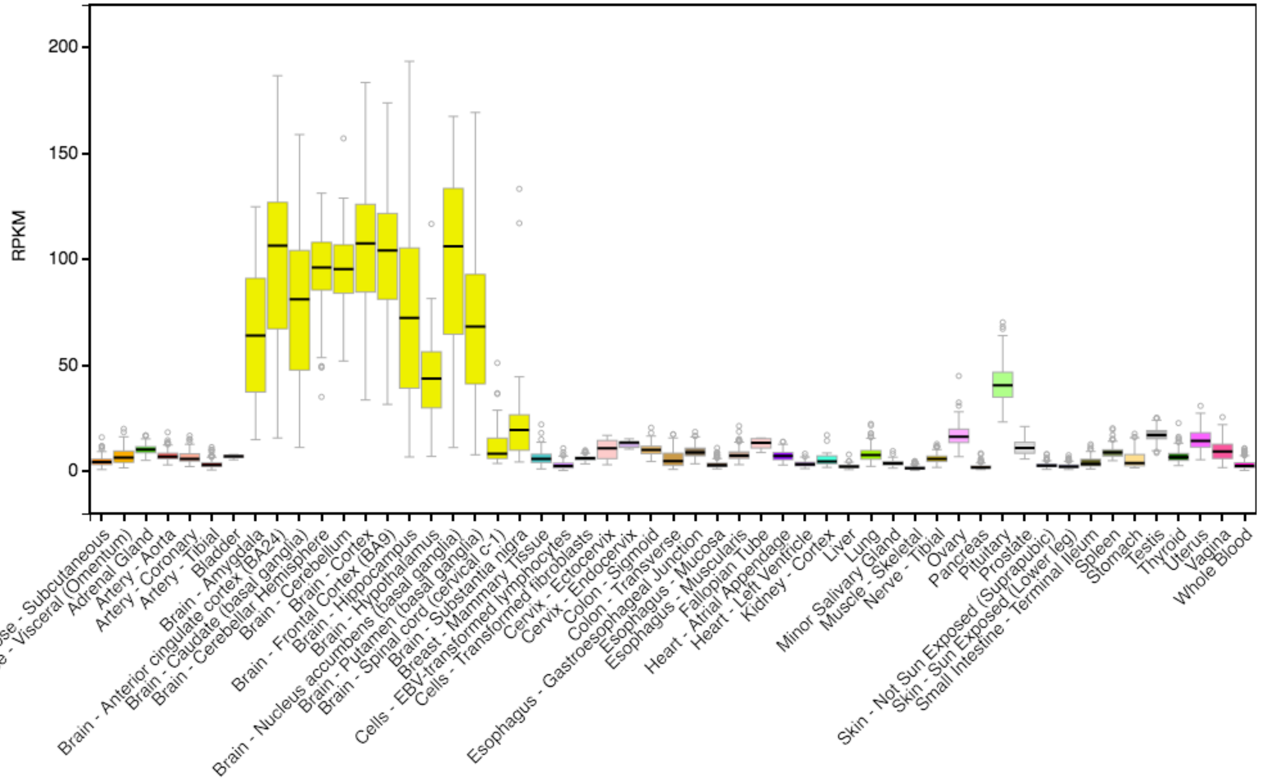

Supplementary Figure 8 Supporting open-access data on cell-type and tissue expression of DLG4.
Panel a): Glia-specific gene interactions within SPN1 (left) and SPN2 (right), reconstructed with high confidence from prior experimental data (GIANT, Greene et al. 2015). Panel b): Expression of DLG4 in different human tissues, extracted from the GTEx Portal (www.gtexportal.org/). Panel c): DLG4 expression in the human brain by region (UKBEC BRAINEAC resource).

Genes coding proteins in SPNs come from all three gene networks. Members involved in development are not involved in IL-1b response.

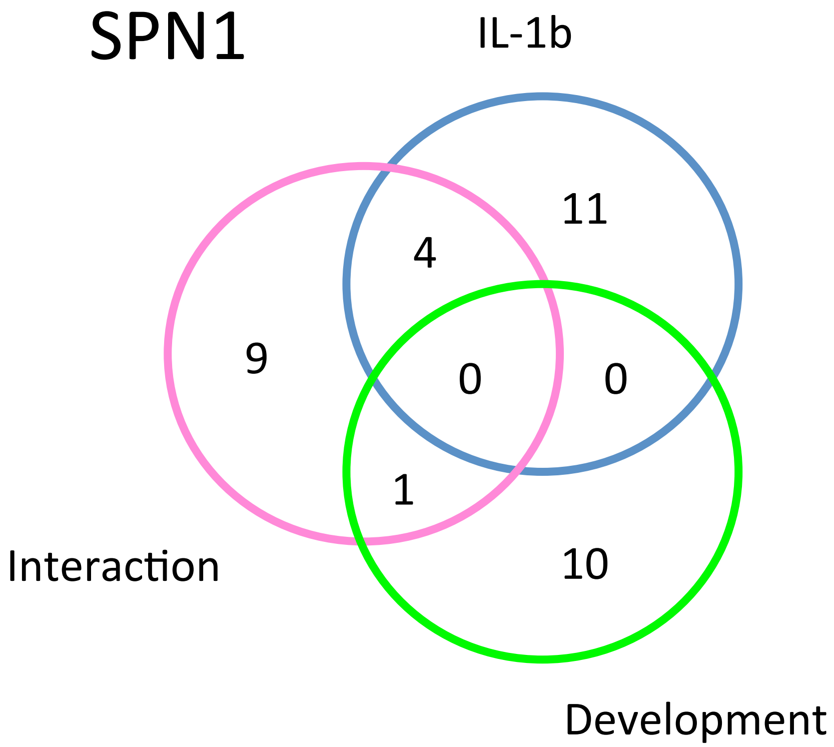

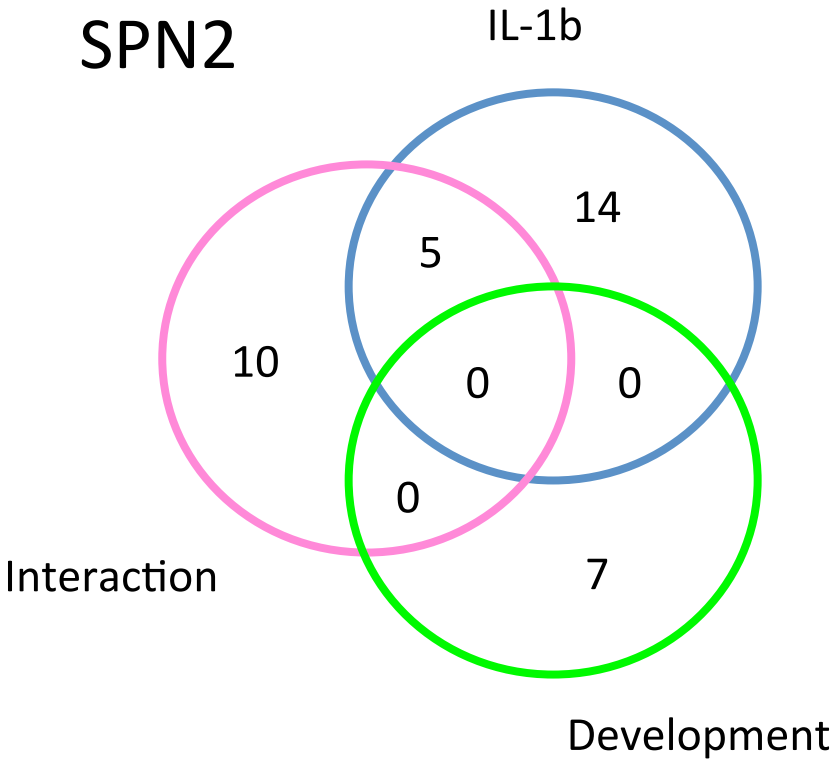

Total	Condition	Genes	Timepoint	Genes2	Interaction	Genes3
**SPN1: 36**	15/1712	Araf, Atp2b2, Arc, Camk2a, Cyfip2, Dlg4, Fah, Gnao1, Ifit3, Kcnj16, Med13, Med18, Med28, Notch1, Slc1a3	11/741	Atp1b2, Cux2, Dlgap3, Kat2a, Kpnb1, Pcbp2, Pdxk, Prrt1, Rps14, Sh3kbp1, Tada3	14/1389	Adam22, Atp2b4, Camk2a, Cyfip2, Gnao1, Kalrn, Ldhb, Nf2, Notch1, PPP1r12a, Rgs12, Rpsa, Shank1, Tada3
**SPN2: 35**	19/1712	Arid1b, Gja1, Gsta4, Hif1a, Hras1, Ksr1, Mapk8, Pdlim5, Prkca, Prkce, Sox17, Src, Srrm2, Ss18l1, Stat3, Tmod1, Tnni3, Tsga10, Wwox	7/741	Ap1b1, Cul4a, Egln1, Lnpep, Raf1, Smarca4, Smarcb1	15/1389	Arnt2, Dvl2, Esr1, Hif1a, Hoxb4, Hras1, Itgb3, Kdm4a, Mapk8, Pbrm1, Slc2a4, Sorbs3, Src, Tnni3, Wwox

Supplementary Figure 9 Gene network membership of SPN proteins.
Genes belonging to more than one network are highlighted in red in the table.

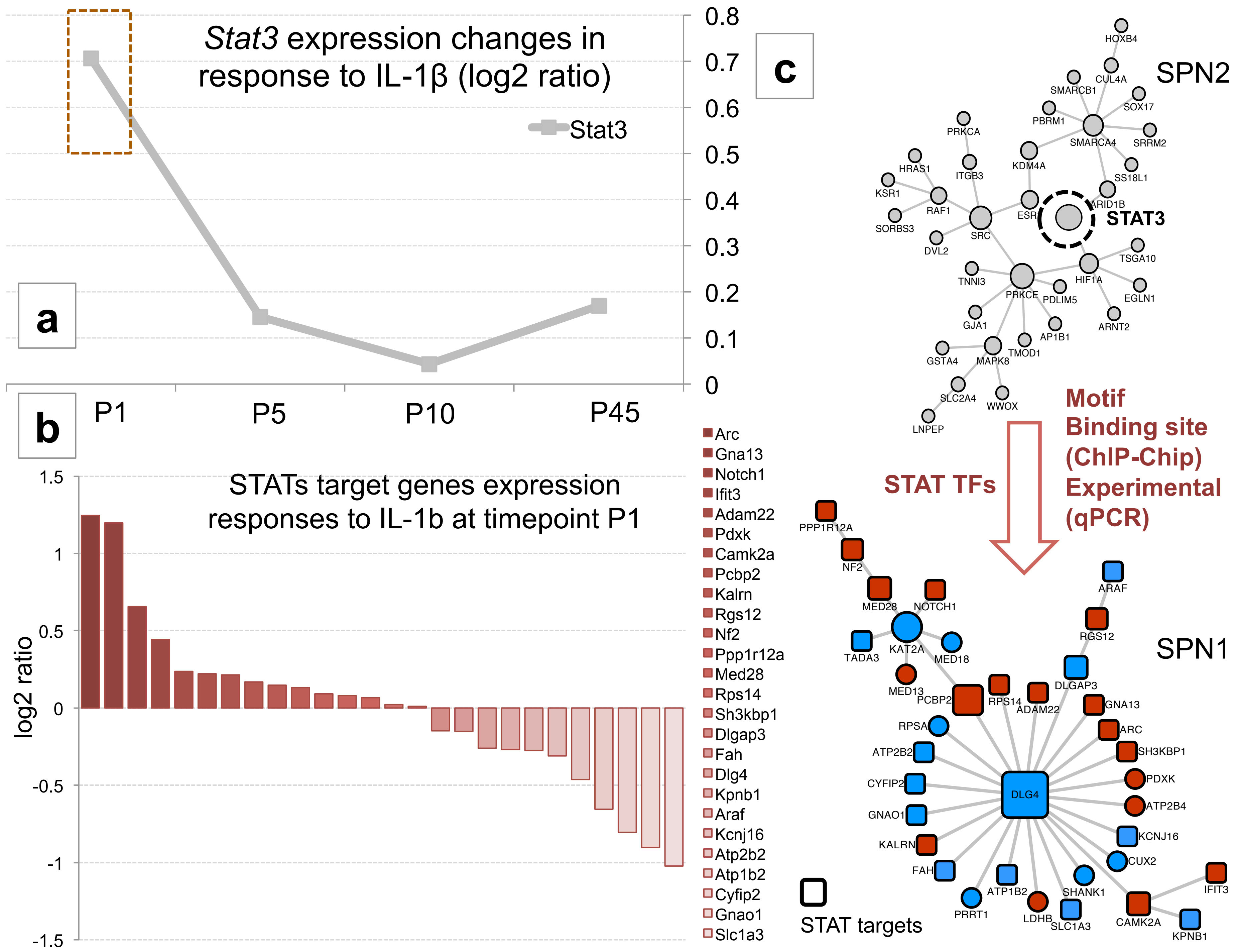

a
Stat3 expression changes in response to IL-1β (log2 ratio)
Stat3
0.8
0.7
0.6
0.5
0.4
0.3
0.2
0.1
0
P1
P5
P10
P45
b
STATs target genes expression responses to IL-1b at timepoint P1
log2 ratio
1.5
1
0.5
0
-0.5
-1
-1.5
Arc
Gna13
Notch1
Ifit3
Adam22
Pdxk
Camk2a
Pcbp2
Kalrn
Rgs12
Nf2
Ppp1r12a
Med28
Rps14
Sh3kbp1
Dlgap3
Fah
Dlg4
Kpnb1
Araf
Kcnj16
Atp2b2
Atp1b2
Cyfip2
Gnao1
Slc1a3
c
SPN2
HOXB4
CUL4A
SMARCB1
SOX17
PBRM1
SMARCA4
SRRM2
PRKCA
KDM4A
SS18L1
HRAS1
ITGB3
ARID1B
KSR1
RAF1
ESR
STAT3
SORBS3
SRC
DVL2
TSGA10
TNNI3
PRKCE
HIF1A
PDLIM5
EGLN1
GJA1
AP1B1
ARNT2
GSTA4
MAPK8
TMOD1
SLC2A4
WWOX
LNPEP
Motif
Binding site
(ChIP-Chip)
Experimental
(qPCR)
STAT TFs
PPP1R12A
NF2
MED28
NOTCH1
ARAF
RGS12
TADA3
KAT2A
MED18
SPN1
MED13
PCBP2
RPS14
ADAM22
DLGAP3
GNA13
RPSA
ARC
ATP2B2
SH3KBP1
CYFIP2
PDXK
DLG4
ATP2B4
GNAO1
KCNJ16
KALRN
CUX2
FAH
ATP1B2
SHANK1
IFIT3
PRRT1
LDHB
SLC1A3
CAMK2A
KPNB1
STAT targets

Supplementary Figure 10 Transcriptional relationships between STAT3 and SPN1.
Panel a) Stat3 transcript level by time-point. Panel b) Transcriptional response of target genes of STATs (STAT1, STAT3 or STAT5) to IL1B exposure (log2 ratio between IL1B and PBS microarray measures). Panel c) STAT3 from SPN2 is a predicted transcription factor for 22/36 (61%) members of SPN1 ($p<0.05$, Supp. Tables 15 and 16), corroborated with ChIP-Chip and qPCR data. Red = up regulated in IL1B versus PBS (control) on microarray, Blue = down regulated in IL1B versus PBS on microarray.

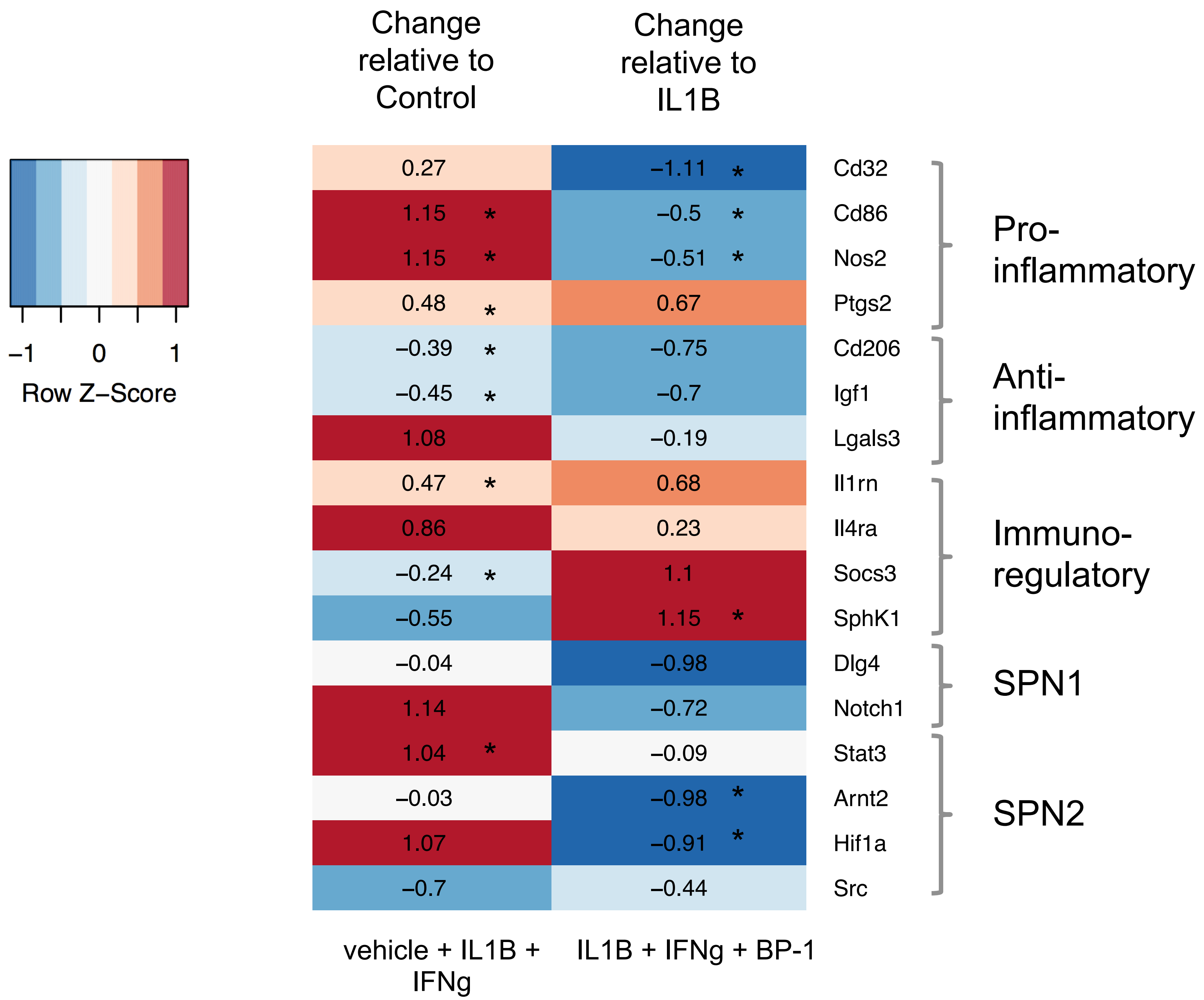
Change relative to Control
Change relative to IL1B
-1
0
1
Row Z-Score
0.27
-1.11 *
Cd32
1.15 *
-0.5 *
Cd86
1.15 *
-0.51 *
Nos2
0.48 *
0.67
Ptgs2
-0.39 *
-0.75
Cd206
-0.45 *
-0.7
Igf1
1.08
-0.19
Lgals3
0.47 *
0.68
Il1rn
0.86
0.23
Il4ra
-0.24 *
1.1
Socs3
-0.55
1.15 *
SphK1
-0.04
-0.98
Dlg4
1.14
-0.72
Notch1
1.04 *
-0.09
Stat3
-0.03
-0.98 *
Arnt2
1.07
-0.91 *
Hif1a
-0.7
-0.44
Src
Pro-inflammatory
Anti-inflammatory
Immuno-regulatory
SPN1
SPN2
vehicle + IL1B + IFNg
IL1B + IFNg + BP-1

Supplementary Figure 11 Effect of STAT3 inhibition (with BP-1) on transcriptional responses to IL1B + IFNg of SPN genes and microglia functional markers.

MACS-isolated primary microglia (P1 mouse) assessed 4 hours after in-vitro IL1B/IFNg exposure. Heatmap with values normalised by row, so each original value is transformed to a row Z-score, i.e. the number of standard deviations above or below the mean of its row. This gives a distribution centred around the midpoint of the colour scale [z = (x - mean)/sd]. Colours represent direction of relative changes (red = increased; blue = decreased). Hierarchical clustering is applied to the rows. Asterisks in the first column represent significant change in IL1B versus Control, and asterisks in the second column represent significant changes in BP-1 versus IL1B exposure (Student t-test, $p < 0.05$, performed using original values). BP-1: small molecule inhibitor of STAT3 (BP-1-102). Vehicle: DMSO (Dimethylsulfoxide, vehicle). IL1B: Interleukin 1 Beta. IFNg: Interferon Gamma. Genes: Notch1: notch 1; Igf1: insulin-like growth factor 1; Socs3: suppressor of cytokine signaling 3; Stat3: signal transducer and activator of transcription 3; Arnt2: aryl hydrocarbon receptor nuclear translocator 2; Hif1a: hypoxia inducible factor 1, alpha subunit; Nos2: nitric oxide synthase 2, inducible; Cd86: Cd86 antigen; Dlg4: discs, large homolog 4 (Drosophila); Src: Rous sarcoma oncogene; Lgals3: lectin, galactose binding, soluble 3; Ptgs2: prostaglandin-endoperoxide synthase 2; Cd32: Fc fragment of IgG, low affinity IIa, receptor; IL4ra: IL-4 Receptor Subunit Alpha; IL1rn: Interleukin 1 Receptor Antagonist; Cd206: Mannose Receptor, C Type 1; Sphk1: Sphingosine Kinase 1.

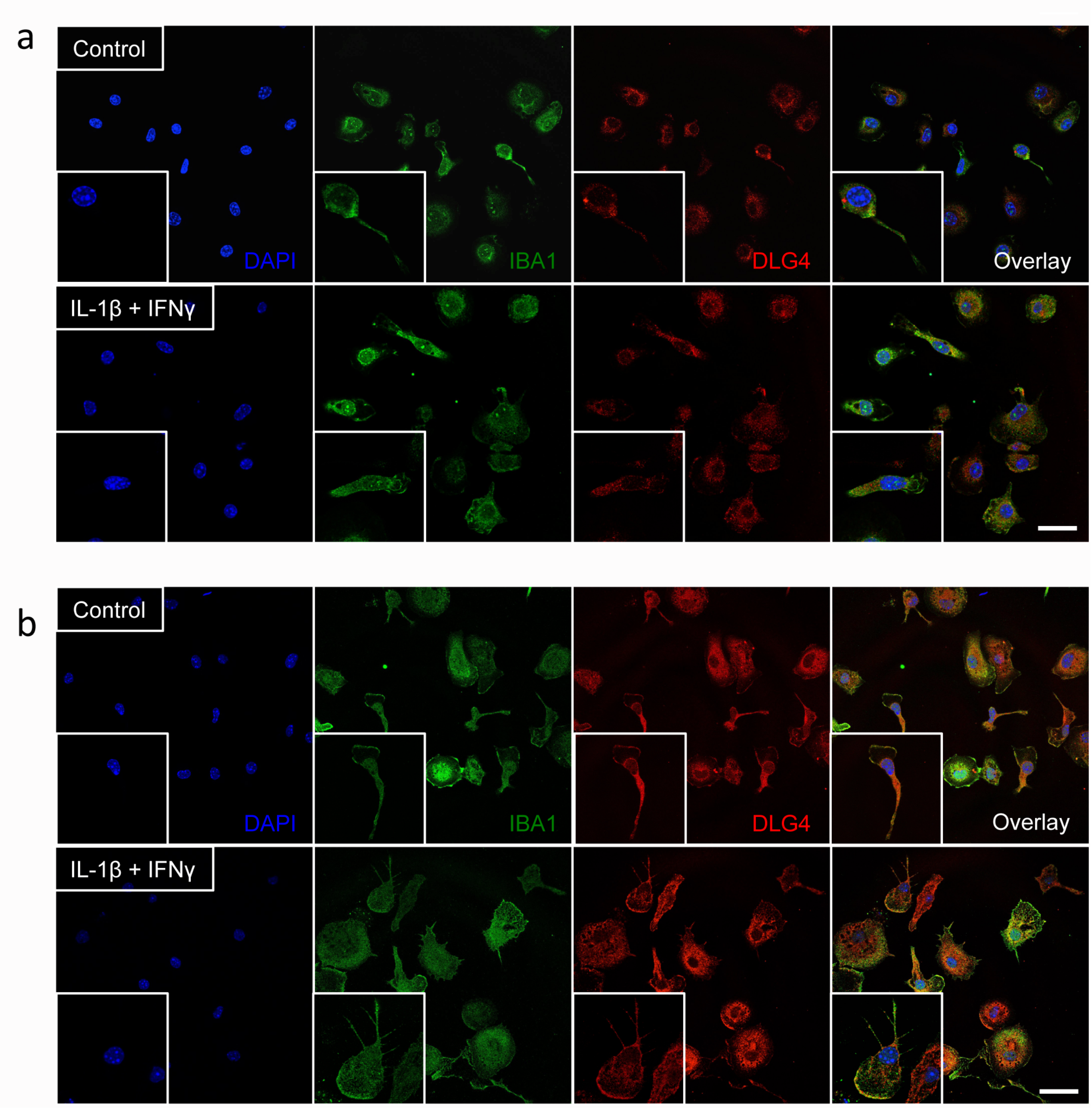
a
Control
DAPI
IBA1
DLG4
Overlay
IL-1β + IFNγ
b
Control
DAPI
IBA1
DLG4
Overlay
IL-1β + IFNγ

Supplementary Figure 12 DLG4 protein is expressed by primary microglia from P1 mice.
All cells were IBA1+ cells. Cells were treated with IL1B + IFNg during 6 hours (50ng/ml and 20ng/ml, respectively). (a). Cells labelled with antibody against DLG4 from Abcam, Scale bar = 40µm. (b) Cells labelled with antibody against DLG4 from Thermofischer, Scale bar = 40µm.

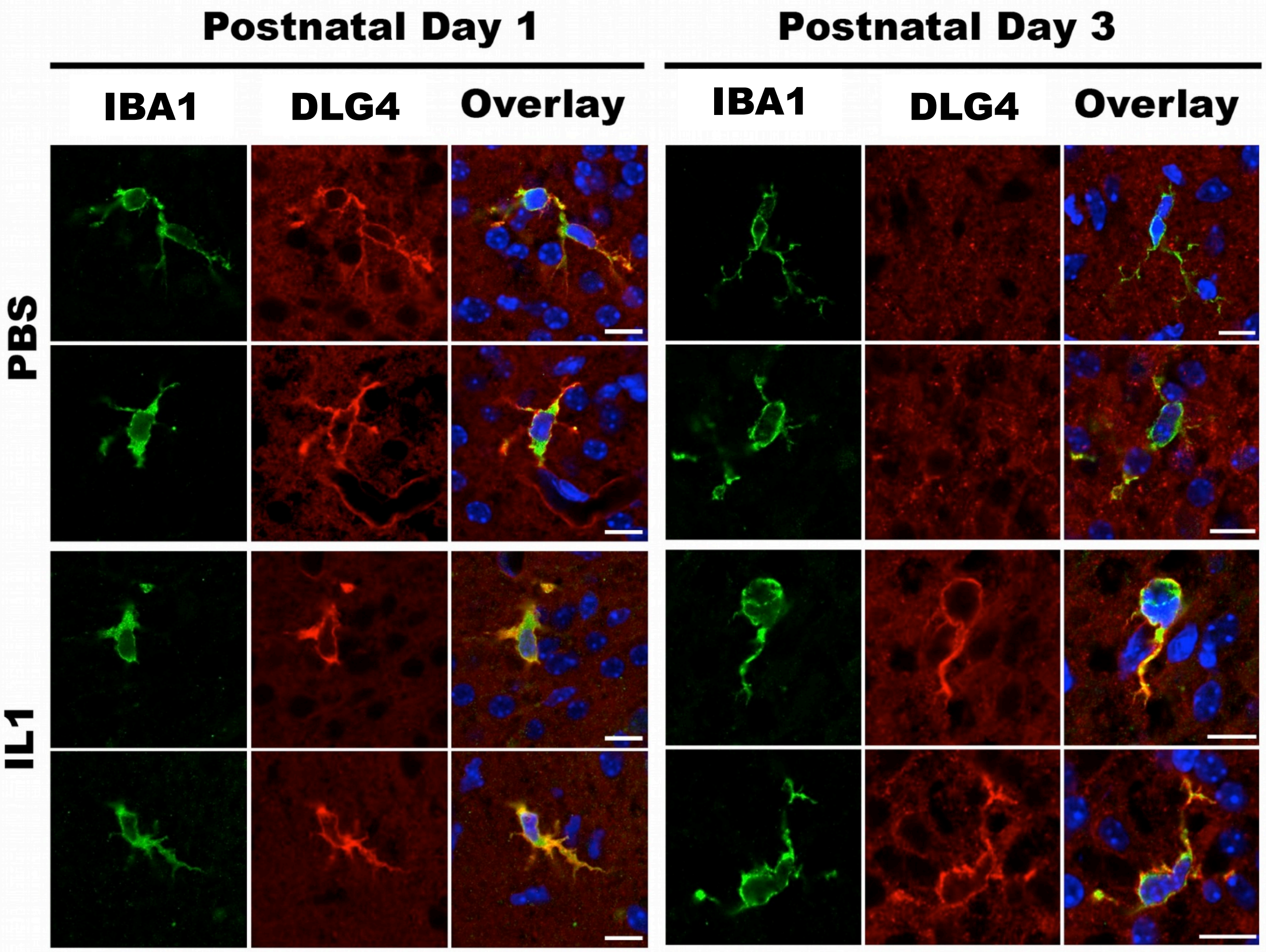
Postnatal Day 1
Postnatal Day 3
IBA1
DLG4
Overlay
IBA1
DLG4
Overlay
PBS
IL1

Supplementary Figure 13 Immunohistochemistry of mouse brain sections at P1 and P3 with/without in-vivo exposure to IL1B. Double labelling of mouse microglia with DLG4 (PSD-95) monoclonal antibodies and a microglial marker (IBA1), under control (PBS) and stimulated (IL1B) conditions, at postnatal day 1 (P1) and P3.

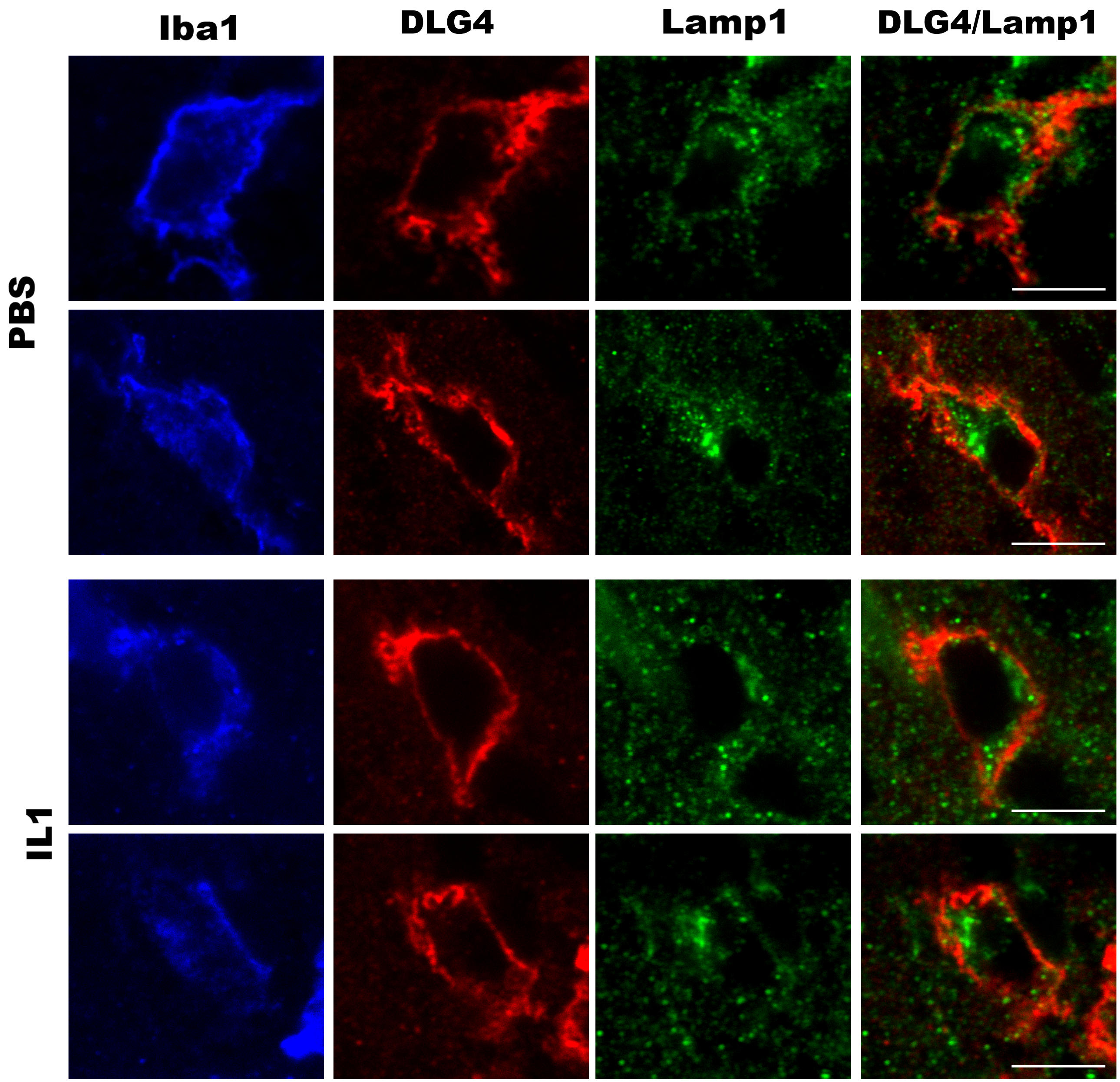
Iba1
DLG4
Lamp1
DLG4/Lamp1
PBS
IL1

Supplementary Figure 14 DLG4 (PSD95) expressed by microglial cells is not localized in lysosomal compartments labelled by LAMP1. In tissue sections from P1 mouse, microglial cells expressing IBA1 (blue panels) from PBS or IL1B treated animals (P1), DLG4 (PSD95) immunoreactivity (red panels) is predominantly located at the surface of cell bodies and ramifications. Conversely, LAMP1 immunofluorescence (green panels) is mainly confined to intracellular vesicles. No colocalisation is thus observed between DLG4 and LAMP1 (DLG4/LAMP-1 panels). Scale bar 5 μm.

DLG4 expression in the human brain through life

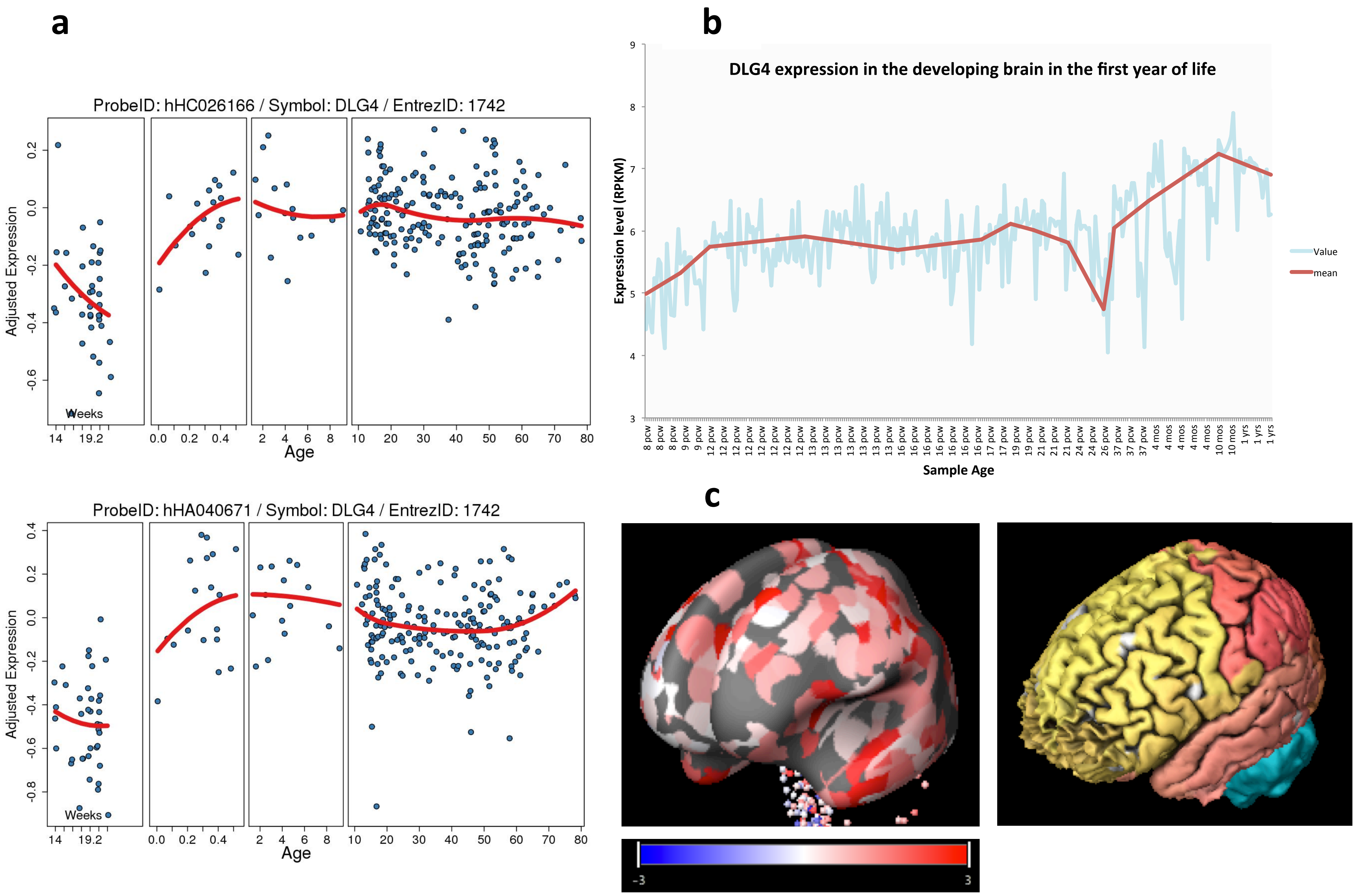

Supplementary Figure 15 Expression of DLG4 in the human brain through life.
Panel a) Temporal dynamics of DLG4 transcription in human prefrontal cortex in an extensive series of post-mortem brains from fetal development through ageing (Brain Cloud). Panel b) DLG4 expression in human tissue brain samples from conception to 1 year of life (Brainspan Developmental Transcriptome). C) DLG4 gene expression mapped to the adult cortex. Samples (either on the inflated surface or as spheres) are color coded by gene expression value as z-score (left). Anatomical surface coloured by brain region (right).

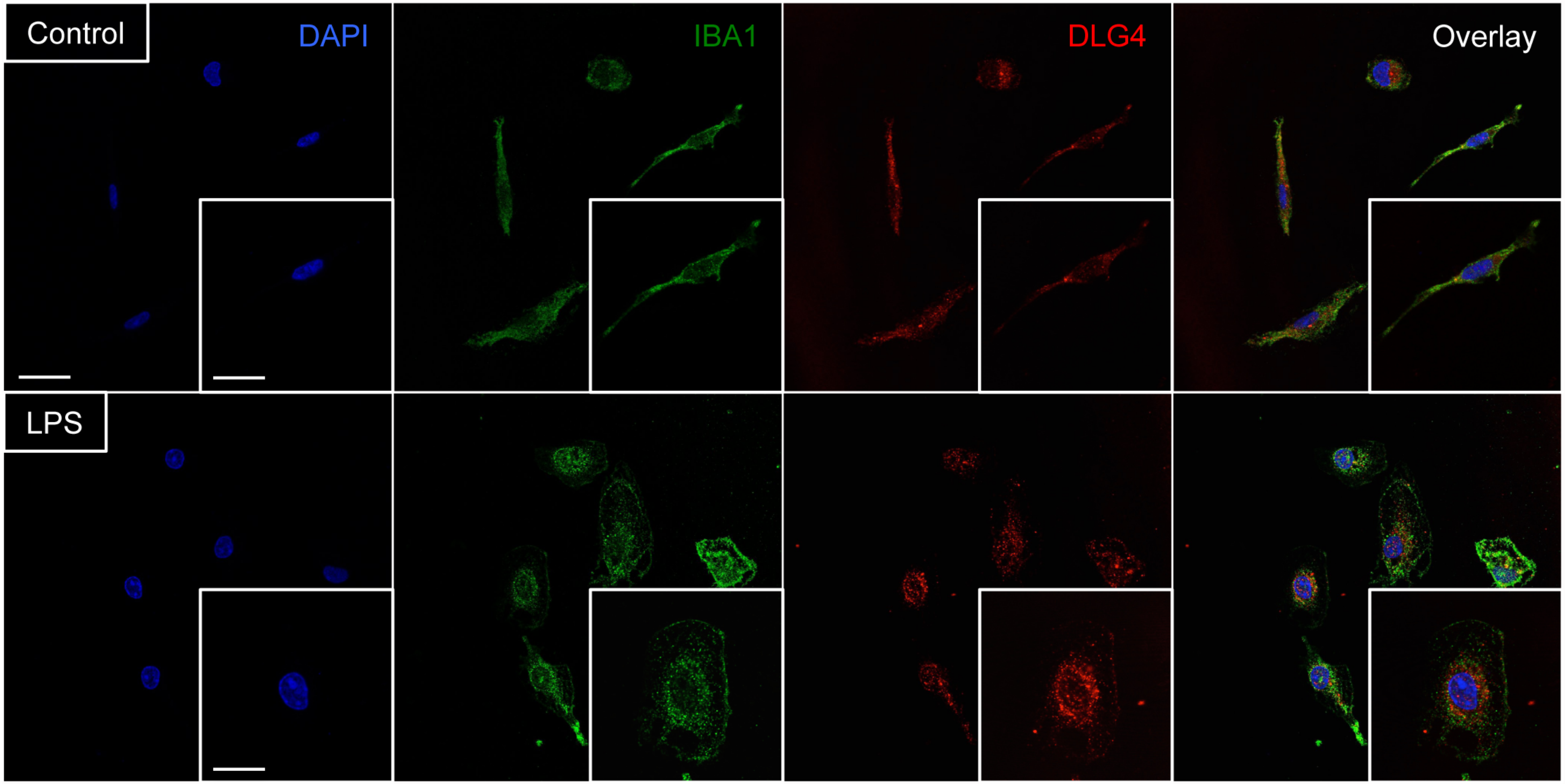
Control
DAPI
IBA1
DLG4
Overlay
LPS

Supplementary Figure 16 DLG4 is expressed by human primary microglia.
Panel a): All cells were IBA1+ cells. LPS (10ng/ml) treatment of IBA1+ human primary microglia during 4 hours induces morphological change. Scale bar = 50µm. Panel b): LPS (10ng/ml) treatment of IBA1+ human primary microglia during 4 hours induces overexpression of pro-inflammatory marker PTGS2 mRNA. n=4/group, mean ±SEM, Mann–Whitney : * $p<0.05$; Panel c): LPS (10ng/ml) treatment of IBA1+ human primary microglia during 4 hours induces down-regulation of CTNNB1 mRNA n=5/group, mean±SEM, Mann–Whitney : ** $p<0.01$.

rs17203281 expression level by genotype

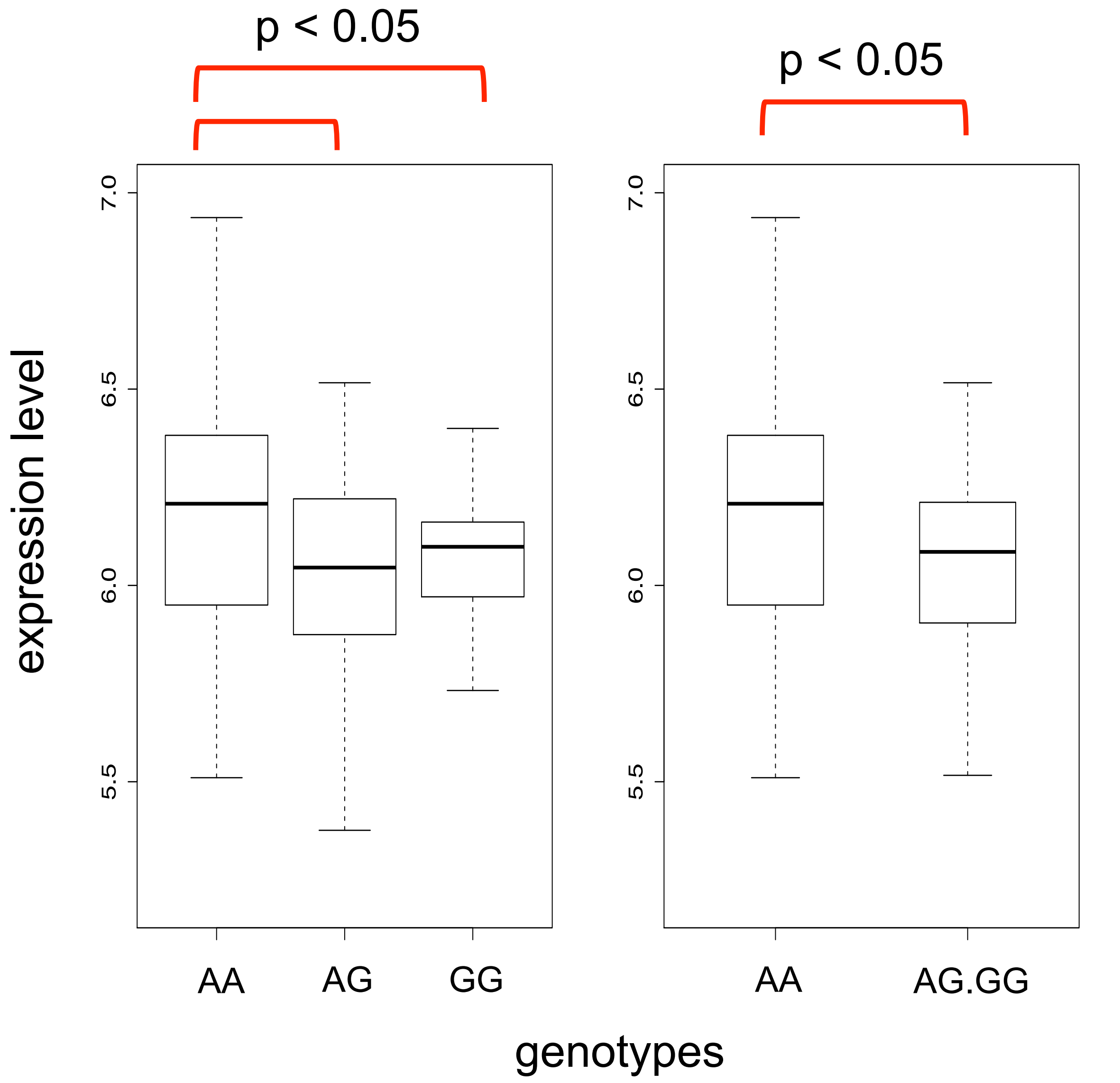

Supplementary Figure 17 cis-eQTL analysis of genotype at DLG4 SNP rs17203281 and DLG4 expression. Expression of DLG4 gene from adult human brain white matter whole tissue (UKBEC repository).

Intersections between SPN1-2 and neuropsychiatry disease modules

1. SPN1
2. SPN2
3. ASD_ID_M1
4. ASD_ID_M2
5. SZ_M1
6. SZ_M2
7. Epilepsy_M1

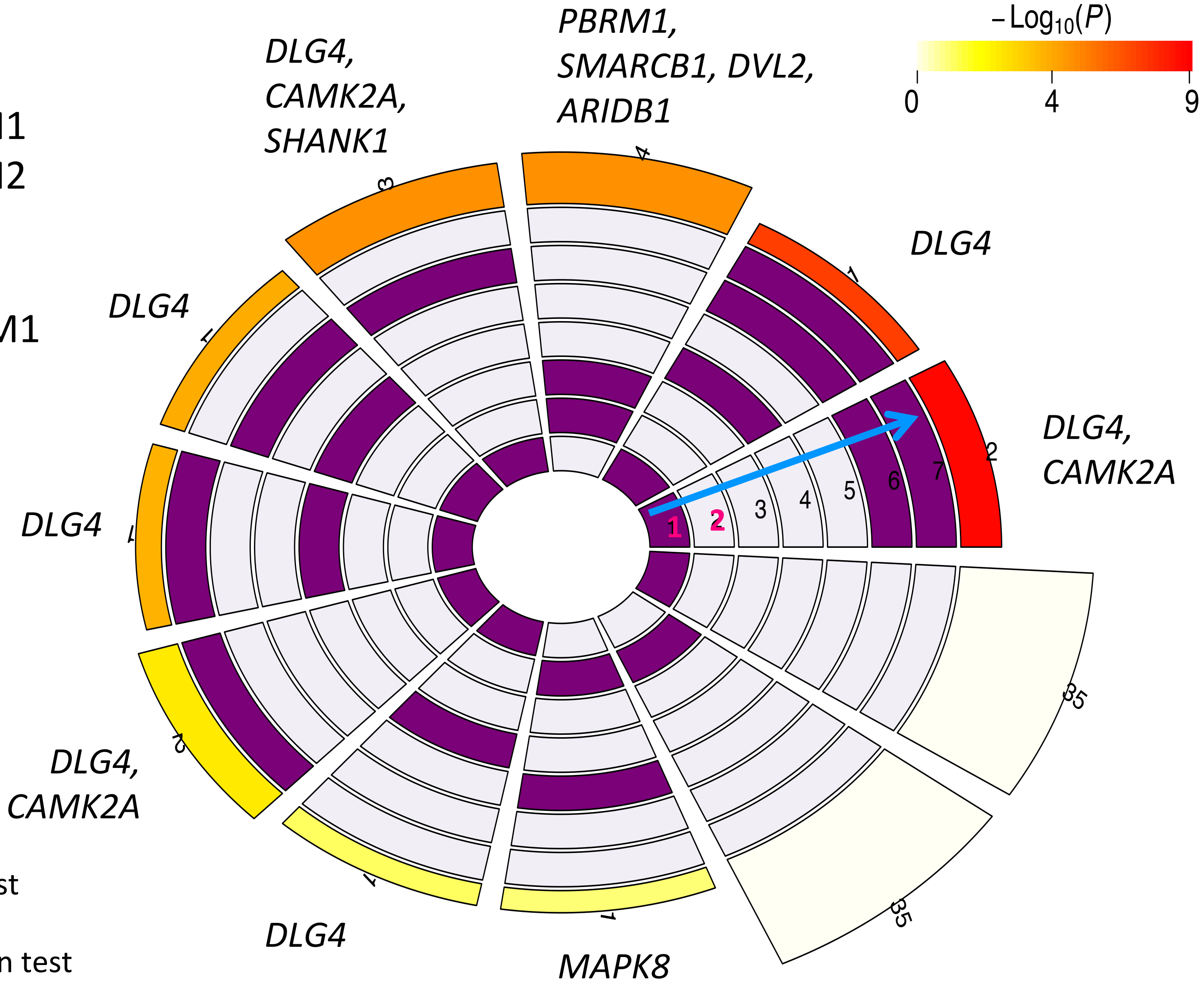

gene set in test

gene set not in test

Supplementary Figure 18 Visualization of the intersections among seven gene lists (SPN1, SPN2 and five gene lists from Hormozdiari et al., Genome Res. 2015.

A circular plot illustrating all possible intersections involving SPN1-2 and the corresponding statistics. The seven tracks in the middle represent the seven gene lists, with individual coloured blocks showing “presence” (dark) or “absence” (light) of the gene lists in each intersection. The height of the bars in the outer layer is proportional to the log of intersection sizes, indicated by the numbers on the top of the bars. The colour intensity of the bars represents the P value significance of the intersections (background = 19,000 protein-coding human genes). The genes contributing to each intersection are shown above each segment. Set codes: SPN1 (super-power node 1); SPN2 (super-power node 2); ASD_ID_M1: Autism Module 1; ASD_ID_M2: Autism Module 2; SZ_M1: Schizophrenia Module 1; SZ_M2: Schizophrenia Module 2; Epilepsy_M1: Epilepsy Module 1. Sets 3:7 extracted from Hormozdiari et al., Genome Res. 2015.

Gene Symbol	Gene Name	EntrezGene	Ensembl
Aasdhppt	aminoadipate-semialdehyde dehydrogenase-phosphopantetheinyl transferase	60496	ENSG00000149313
Cdc42Ep2	CDC42 effector protein (Rho GTPase binding) 2	10435	ENSG00000149798
Fblim1	filamin binding LIM protein 1	54751	ENSG00000162458
Glo1	glyoxalase I	2739	ENSG00000124767
Ick	intestinal cell (MAK-like) kinase	22858	ENSG00000112144
Mkl2	MKL/myocardin-like 2	57496	ENSG00000186260
Mmab	methylmalonic aciduria (cobalamin deficiency) cblB type	326625	ENSG00000139428
Mphosph9	M-phase phosphoprotein 9	10198	ENSG00000051825
Mtap7D3	MAP7 domain containing 3	320923	ENSMUSG00000067878
Nhsl2	NHS-like 2	340527	ENSG00000204131
P2Rx7	purinergic receptor P2X, ligand-gated ion channel, 7	5027	ENSG00000089041
Ppp1R7	protein phosphatase 1, regulatory subunit 7	5510	ENSG00000115685
Prtn3	proteinase 3	5657	ENSG00000196415
Psme2	proteasome (prosome, macropain) activator subunit 2 (PA28 beta)	5721	ENSG00000100911
Samd9L	sterile alpha motif domain containing 9-like	219285	ENSG00000177409
Sp110	SP110 nuclear body protein	3431	ENSG00000135899
Tgs1	trimethylguanosine synthase 1	96764	ENSG00000137574
Tmem218	transmembrane protein 218	219854	ENSG00000150433
Tspan32	tetraspanin 32	10077	ENSG00000064201
Whsc1L1	Wolf-Hirschhorn syndrome candidate 1-like 1	54904	ENSG00000147548
Xaf1	XIAP associated factor 1	54739	ENSG00000132530
Zdhhc21	zinc finger, DHHC-type containing 21	340481	ENSG00000175893

Supplementary Table 1. List of genes present in all three gene co-expression networks (IL1B, Development and Interaction).

Network	Interaction	Development	IL1B
**Clustering Coefficient**	0.251	0.354	0.277
**Characteristic path length**	3.542	5.739	3.133
**Avg. no. neighbours**	8.119	3.819	13.8
**No. of nodes**	1406	663	1764
**Node degree exponent**	-1.6	-1.85	-1.44
**Power law R squared**	0.916	0.887	0.886

Supplementary Table 2. Summary topological features of gene networks, calculated with Cytoscape plugin NetworkAnalyzer.

Clustering coefficient: ratio *N* / *M*, where *N* is the number of edges between the neighbours of *n*, and *M* is the maximum number of edges that could possibly exist between the neighbours of *n*.

Characteristic path length: expected distance between two connected nodes.

Average number of neighbours: average connectivity of a node in the network (the size of its neighbourhood)

No. of nodes: number of genes in the network

Node degree exponent: Scale-free networks are characterized by a power-law degree distribution; the probability that a node has *k* links follows $P(k) \sim k^{-\gamma}$, where γ is the degree exponent.

Power law R squared: the proportion of variability in a data set, which is explained by a fitted linear model. Therefore, the R-squared value is computed on logarithmized data, where the power-law curve: $y = \beta x^{\alpha}$ is transformed into linear model: $\ln y = \ln \beta + \alpha \ln x$

Network	GO annotation (adj. p < 0.01)
**SPN1**	regulation of molecular function, negative regulation of muscle cell differentiation, cellular metabolic process, organic substance metabolic process, social behaviour, vocalisation behaviour, neuromuscular process, protein domain specific binding, glutamate receptor binding, scaffold protein binding, structural molecule activity, mediator complex, ionotropic glutamate receptor complex, extrinsic to internal side of plasma membrane, cell projection part, neuron projection, synapse
**SPN2**	positive regulation of cellular biosynthetic process, positive regulation of nucleobase-containing compound metabolic process, positive regulation of gene expression, system development, transcription regulatory process, protein kinase binding, steroid hormone receptor binding, transcription regulatory region DNA binding, signal transducer activity, cytosol, SWI/SNF complex

Supplementary Table 3. Gene Ontology (GO) annotation for the two super-power nodes (SPN1 and SPN2), using the WebGestalt platform. Benjamini-Hochberg adjusted P values reported.

SPN1	Disease	Gene name	Statistics
	Epilepsy	SLC1A3, ARC, ADAM22	adjP=0.0404
	Nervous System Diseases	SLC1A3, ARC, ADAM22, NF2, ATP2B2	adjP=0.0404
	Myotonic Dystrophy	PPP1R12A, PCBP2	adjP=0.0404
	Mental Disorders	DLG4, KALRN, DLGAP3, SLC1A3, CAMK2A	adjP=0.0404
	Neurofibromatosis 2	NF2, MED28	adjP=0.0404
	Brain Diseases	SLC1A3, ARC, ADAM22, FAH	adjP=0.0404
	Tic Disorders	DLGAP3, SLC1A3	adjP=0.0404
	Shell shock	ATP2B2, ATP2B4	adjP=0.0308
	Monosomy	NF2, RPS14	adjP=0.0404
	Paralysis	PPP1R12A, SLC1A3	adjP=0.0404
**SPN2**	**Disease**		
	Drug interaction with drug	ITGB3, PRKCA, PRKCE, MAPK8, RAF1, SRC, ESR1, STAT3	adjP=7.87e-05
	Drug Resistance	ESR1, HIF1A, MAPK8, SLC2A4, STAT3	adjP=0.0028
	Sarcoma	HIF1A, ITGB3, SMARCB1, SRC	adjP=0.0028
	Metaplasia	HIF1A, ITGB3, EGLN1, STAT3	adjP=0.0033
	Neovascularization, Pathologic	HIF1A, ITGB3, EGLN1, STAT3	adjP=0.0033
	Cell Transformation, Neoplastic	WWOX, RAF1, SMARCA4, SRC, STAT3	adjP=0.0047
	cancer or viral infections	ESR1, HIF1A, WWOX, MAPK8, RAF1, SMARCB1, SRC	adjP=0.0047
	Sarcoma, Kaposi	HIF1A, ITGB3, STAT3	adjP=0.0055
	Sarcoma, Avian	PRKCA, SRC	adjP=0.0055
	Stomach Neoplasms	HIF1A, WWOX, SOX17, STAT3	adjP=0.0060

Supplementary Table 4. Disease term enrichment for super-power nodes (SPN1 and SPN2), using the Webgestalt tool as before.

Transcription Factor	Matrix	Association Score	P-Value
**Spz1**	SPZ1_01	3.647	2.50E-03
**Hic-1**	HIC1_02	3.527	3.10E-03
**Roaz**	ROAZ_01	3.421	3.91E-03
**Vdr**	VDR_Q6	2.915	1.09E-02
**Ap-2alpha , Ap-2alphaa**	AP2_Q3	2.766	1.42E-02
**Pax-5**	PAX5_01	2.726	1.65E-02
**Stat3**	STAT3_02	2.687	1.76E-02
**Zf5**	ZF5_01	2.496	2.44E-02
**Atf-1**	ATF1_Q6	2.489	2.47E-02
**Coup-tf1 , Hnf-4alpha1**	COUP_01	2.384	3.13E-02
**Err1**	ERR1_Q2	2.363	3.22E-02
**Hnf-1alpha , Hnf-1alpha-a**	HNF1_Q6_01	2.293	3.61E-02
**Sp1 , Sp3**	SP1_Q6_01	2.29	3.64E-02
**Pou3f2**	BRN2_01	2.251	3.80E-02
**Stat6**	STAT6_02	2.211	4.24E-02
**Elf-1 , Elf-1**	ELF1_Q6	2.183	4.51E-02
**Pu.1**	PU1_Q6	2.183	4.51E-02
**Rar-alpha1 , Rar-beta**	T3R_Q6	2.183	4.51E-02
**Stat1alpha**	STAT1_02	2.137	4.81E-02
**Ap-2 , Ap-2alpha**	AP2_Q6	2.073	5.52E-02

Supplementary Table 5. Predicted transcription factors for SPN1 using PASTAA tool, Roider et al, 2009.

Rank	Gene ID	Alternate Gene Synonym	Affinity Score	Top Regulating Factors
159	ENSMUSG00000043861	Atp1b2	0.2533	[ELF1_Q6, PU1_Q6, OCT_C, SRF_C, & P_01]
225	ENSMUSG00000024608	Rps14	0.235	[PAX6_Q2, HMX1_01, PAX_Q6, RORA2_01, & ABF1_01]
5177	ENSMUSG00000030630	Fah	0.1216	[SREBP_Q3, HNF4ALPHA_Q6, ZIC1_01, CDP_01, & SREBP1_02]
5436	ENSMUSG00000009073	Nf2	0.12	[PAX6_Q2, PAX_Q6, FOXP1_01, NRF2_01, & WHN_B]
6533	ENSMUSG00000051497	Kcnj16	0.1123	[EVI1_02, TITF1_Q3, IRF2_01, ZID_01, & T3R_01]
6697	ENSMUSG00000062488	Ifit3	0.1104	[AREB6_04, LDSPOLYA_B, SMAD3_Q6, SMAD_Q6_01, & IRF7_01]
8838	ENSMUSG00000001127	Araf	0.09917	[CHX10_01, SRF_Q5_02, SRF_01, YY1_Q6_02, & SRF_C]
10661	ENSMUSG00000020340	Cyfip2	0.09213	[RAP1_C, SREBP1_02, GRE_C, CDC5_01, & BZIP910_01]
11263	ENSMUSG00000040990	Sh3kbp1	0.08949	[PIF3_01, REPCAR1_01, HSF_02, ABI4_01, & HSF_02]
11598	ENSMUSG00000056851	Pcbp2	0.08787	[HMEF2_Q6, PAX6_Q2, PAX_Q6, MEF2_01, & MTATA_B]
12005	ENSMUSG00000005360	Slc1a3	0.08557	[GAGAFACTOR_Q6, FTZ_01, PAX6_Q2, PAX4_01, & HSF_02]
13587	ENSMUSG00000026923	Notch1	0.07747	[GAGAFACTOR_Q6, SREBP_Q3, SKN1_01, E2_Q6, & HSF1_Q6]
16569	ENSMUSG00000032788	Pdxk	0.06807	[HSF_03, AR_01, ARNT_02, USF_01, & CG1_Q6]
17289	ENSMUSG00000001440	Kpnb1	0.06569	[MYCMAX_01, HEN1_02, SPZ1_01, DR3_Q4, & CAAT_C]
17523	ENSMUSG00000040537	Adam22	0.06487	[AP1_01, R_01, GCM_Q2, GCN4_01, & ETS_Q4]
18619	ENSMUSG00000019907	Ppp1r12a	0.06064	[ZAP1_01, HOX13_01, NRF1_Q6, GAGAFACTOR_Q6, & ER_Q6]
21506	ENSMUSG00000024617	Camk2a	0.04638	[RFX1_02, MEIS1BHOXA9_02, RFX_Q6, EFC_Q6, & ACAAT_B]
23095	ENSMUSG00000029101	Rgs12	0.04095	[LFA1_Q6, ABI4_01, PCF2_01, MAZR_01, & HSF_04]
24214	ENSMUSG00000022602	Arc	0.03695	[E2A_Q2, SRF_C, SRF_Q4, SRF_Q5_02, & SRF_Q6]
24773	ENSMUSG00000030302	Atp2b2	0.03451	[SF1_Q6, HSF_Q6, HOX13_01, SREBP_Q3, & ERR1_Q2]
26417	ENSMUSG00000031748	Gnao1	0.01865	[CREB_Q2_01, ATF3_Q6, MYCMAX_B, CREB_Q4_01, & CREB_Q2]
26899	ENSMUSG00000020611	Gna13	0.01518	[ISRE_01, PR_01, USF_Q6_01, PAX5_02, & TFIII_Q6]

Supplementary Table 6. Predicted targets of STAT3 in SPN1 based on PASTAA tool as before.

Matrix	Transcription Factor	Association Score	P-Value
**ZF5_01**	Zf5	3.854	1.75E-03
**MZF1_02**	Mzf-1	3.751	2.14E-03
**EGR1_01**	Egr-1	3.702	2.34E-03
**HIF1_Q3**	Hif-1alpha	3.592	2.99E-03
**CMAF_01**	C-maf	3.467	3.67E-03
**HIF1_Q5**	Hif-1alpha	3.286	5.53E-03
**XBP1_01**	Xbp-1	3.191	6.44E-03
**ZF5_B**	Zf5	3.113	7.57E-03
**AP2_Q6**	Ap-2 , Ap-2alpha	3.066	8.11E-03
**E2F_Q2**	Dp-1 , E2f-1	2.956	1.09E-02
**MYOGENIN_Q6**	Myogenin	2.822	1.40E-02
**E2F1_Q3**	E2f-1	2.782	1.49E-02
**AP2ALPHA_01**	Ap-2alpha , Ap-2alphaa	2.767	1.53E-02
**COUPTF_Q6**	Coup , Coup-tf1	2.726	1.68E-02
**E47_02**	E47	2.726	1.76E-02
**AP1_Q4_01**	Fosb , Fra-1	2.716	1.80E-02
**HEB_Q6**	Heb	2.691	1.87E-02
**EGR3_01**	Egr-3	2.666	1.94E-02
**SREBP1_Q6**	Srebp-1 , Srebp-1c	2.653	1.99E-02
**AP2GAMMA_01**	Ap-2gamma	2.6	2.10E-02
**AP4_Q6**	Ap-4	2.552	2.32E-02
**TAXCREB_01**	Creb , Deltacreb	2.496	2.60E-02
**AP1_Q6_01**	Fosb , Fra-1	2.479	2.67E-02
**BACH2_01**	Bach2	2.479	2.67E-02
**AP4_Q6_01**	Ap-4	2.475	2.72E-02
**VBP_01**	Vbp	2.475	2.72E-02
**COUP_DR1_Q6**	Coup-tf1 , Coup-tf2	2.425	3.06E-02
**HNF4_01**	Hnf-4alpha1 , Hnf-4alpha2	2.425	3.06E-02
**HNF4_DR1_Q3**	Hnf-4 , Hnf-4alpha	2.425	3.06E-02
**VDR_Q6**	Vdr	2.425	3.06E-02
**AHRARNT_02**	Ahr , Arnt	2.339	3.53E-02
**LMO2COM_01**	Lmo2	2.328	3.67E-02
**AP1_01**	Fosb , Fra-1	2.309	3.80E-02
**EGR2_01**	Egr-2	2.306	3.80E-02
**ARNT_02**	Arnt	2.285	3.88E-02
**AP1_Q2_01**	Fosb , Fra-1	2.268	4.01E-02
**COUP_01**	Coup-tf1 , Hnf-4alpha1	2.25	4.27E-02
**DR1_Q3**	Coup-tf1 , Coup-tf2	2.25	4.27E-02
**HNF4_01_B**	Hnf-4alpha1	2.25	4.27E-02
**CP2_02**	Cp2 , Cp2a	2.227	4.45E-02
**NGFIC_01**	Egr-4	2.186	4.62E-02
**CREB_Q3**	Atf-1 , Atf-2	2.171	4.90E-02

Supplementary Table 7. Predicted transcription factors for SPN2 based on PASTAA tool as before.

Antibody	Gene name	Ensembl ID	Chromosome	seq_start	seq_end	score
P-STAT1	Ifit3	ENSRNOG00000022839	1	238567334	238567768	1.56
P-STAT1	Dlg4	ENSRNOG00000018526	10	56862727	56863761	1.43
P-STAT1	Kcnj16	ENSRNOG00000004713	10	100511557	100511901	1.06
P-STAT1	Med28	ENSRNOG00000003592	14	70679043	70679502	1.32
P-STAT1	Slc1a3	ENSRNOG00000016163	2	58348062	58348406	1.16
P-STAT1	Dlgap3	ENSRNOG00000014302	5	146637741	146638077	1.01
P-STAT3	Ifit3	ENSRNOG00000022839	1	238571356	238571710	1.34
P-STAT3	Kalrn	ENSRNOG00000001706	11	68192434	68193063	1.11
P-STAT3	Kalrn	ENSRNOG00000001706	11	68195985	68196324	2.72
P-STAT5	Dlgap3	ENSRNOG00000014302	5	146635248	146635581	1.7
P-STAT5	Sh3kbp1	ENSRNOG00000004322	X	56423927	56424781	1.33

Supplementary Table 8. ChIP-Chip binding peaks for SPN1 genes with STATs (Przanowski et al, 2014).

Cohort	Number of individuals	GA min	GA max	GA mean	PMA min	PMA max	PMA mean
**Pilot**	70	23.29	32.86	28.575139	27.71	52.57	38.66875
**ePrime**	271	23.57	32.86	28.215	38.29	58.29	42.63885609

Supplementary Table 9. Features of preterm clinical cohorts. GA: gestational age; PMA: Post-menstrual age; ages in weeks.

dbSNP rsID	Chromosome position	mRNA position	Heterozygosity	MAF	Function	Protein residue	Codon position	Amino acid
**rs17203281**	7196492	2497	0.371	0.246	synonymous	Ile [I]	3	432
**rs929229**	7202080		0.457	0.3526		intron		
**rs739669**	7219058	993	0.421	0.3007		5' UTR	T	
**rs390200**	7206676		0.5	0.4898		intron		
**rs3826408**	7197973		0.474	0.3866		intron		
**rs2017365**	7219305	746	0.5	0.499		5' UTR	T	
**rs1875673**	7205187		0.429	0.3019		intron		

Supplementary Table 10. DLG4 SNPs on Illumina HumanOmniExpress array (queried in dbSNP), with descriptive features.

Gencode Id	Gene Symbol	SNP Id	P-Value	Effect Size	Tissue
ENSG00000132535.14	DLG4	rs113086489	9.70E-12	0.36	Muscle - Skeletal
ENSG00000132535.14	DLG4	rs390200	6.40E-10	0.3	Muscle - Skeletal
ENSG00000132535.14	DLG4	rs507506	1.00E-09	0.3	Muscle - Skeletal
ENSG00000132535.14	DLG4	rs446994	1.40E-09	0.3	Muscle - Skeletal
ENSG00000132535.14	DLG4	rs222852	2.00E-09	-0.3	Muscle - Skeletal
ENSG00000132535.14	DLG4	rs35224044	2.10E-09	0.3	Muscle - Skeletal
ENSG00000132535.14	DLG4	rs222857	2.70E-09	0.31	Muscle - Skeletal
ENSG00000132535.14	DLG4	rs2428362	2.80E-09	0.31	Muscle - Skeletal
ENSG00000132535.14	DLG4	rs367181	3.00E-09	0.31	Muscle - Skeletal
ENSG00000132535.14	DLG4	chr17_7123240_I	4.10E-09	0.3	Muscle - Skeletal
ENSG00000132535.14	DLG4	chr17_7116398_D	4.50E-09	0.3	Muscle - Skeletal
ENSG00000132535.14	DLG4	rs11867639	5.30E-09	0.29	Muscle - Skeletal
ENSG00000132535.14	DLG4	rs385313	7.80E-09	0.28	Muscle - Skeletal
ENSG00000132535.14	DLG4	rs112216599	8.70E-09	-0.55	Muscle - Skeletal
ENSG00000132535.14	DLG4	rs5418	1.20E-08	0.29	Muscle - Skeletal
ENSG00000132535.14	DLG4	rs178471	1.50E-08	0.28	Muscle - Skeletal
ENSG00000132535.14	DLG4	rs5417	2.10E-08	0.29	Muscle - Skeletal
ENSG00000132535.14	DLG4	rs117395618	4.60E-08	-0.7	Muscle - Skeletal
ENSG00000132535.14	DLG4	rs72837700	5.10E-08	-0.7	Muscle - Skeletal
ENSG00000132535.14	DLG4	rs72839704	5.60E-08	-0.69	Muscle - Skeletal
ENSG00000132535.14	DLG4	chr17_7116413_D	6.10E-08	0.28	Muscle - Skeletal
ENSG00000132535.14	DLG4	rs141804853	7.40E-08	-0.69	Muscle - Skeletal
ENSG00000132535.14	DLG4	rs9797278	1.00E-07	0.3	Muscle - Skeletal
ENSG00000132535.14	DLG4	rs222836	1.20E-07	0.27	Muscle - Skeletal
ENSG00000132535.14	DLG4	rs72839706	1.30E-07	-0.68	Muscle - Skeletal
ENSG00000132535.14	DLG4	rs41283399	1.40E-07	-0.68	Muscle - Skeletal
ENSG00000132535.14	DLG4	rs125651	1.40E-07	0.26	Muscle - Skeletal
ENSG00000132535.14	DLG4	chr17_7116420_I	1.60E-07	-0.61	Muscle - Skeletal
ENSG00000132535.14	DLG4	rs41283401	1.60E-07	-0.67	Muscle - Skeletal
ENSG00000132535.14	DLG4	rs41283403	1.60E-07	-0.67	Muscle - Skeletal
ENSG00000132535.14	DLG4	chr17_7096707_D	2.10E-07	0.26	Muscle - Skeletal
ENSG00000132535.14	DLG4	rs55742293	2.50E-07	-0.77	Muscle - Skeletal
ENSG00000132535.14	DLG4	rs408315	7.90E-07	-0.5	Muscle - Skeletal
ENSG00000132535.14	DLG4	rs3826408	0.0000012	0.24	Muscle - Skeletal
ENSG00000132535.14	DLG4	rs2242449	0.0000014	0.25	Muscle - Skeletal
ENSG00000132535.14	DLG4	rs55868524	0.0000058	0.25	Muscle - Skeletal
ENSG00000132535.14	DLG4	rs222851	0.0000065	-0.24	Muscle - Skeletal
ENSG00000132535.14	DLG4	chr17_7170481_D	0.0000072	0.25	Muscle - Skeletal
ENSG00000132535.14	DLG4	rs12601936	0.0000073	0.24	Muscle - Skeletal
ENSG00000132535.14	DLG4	rs1634381	0.0000088	0.24	Muscle - Skeletal

Supplementary Table 11. Significant known eQTLs in DLG4, retrieved from GTEx portal.

Intersections	Degree	Observed.Overlap	Expected.Overlap	Fold Enrichmment	P.value	Elements
SPN1 & SZ_M2 & Epilepsy_M1	3	2	9.07479E-05	22039.07204	3.73947E-09	CAMK2A, DLG4
SPN1 & ASD_ID_M2 & SZ_M2 & Epilepsy_M1	4	1	1.14629E-07	8723799.349	1.14629E-07	DLG4
SPN2 & ASD_ID_M1	2	4	0.147368421	27.14285714	1.38141E-05	PBRM1, SMARCB1, DVL2, ARID1B
SPN1 & SZ_M2	2	3	0.047894737	62.63736264	1.44607E-05	CAMK2A, DLG4, SHANK1
SPN1 & ASD_ID_M2 & SZ_M2	3	1	6.04986E-05	16529.30403	6.0497E-05	DLG4
SPN1 & ASD_ID_M2 & Epilepsy_M1	3	1	8.37673E-05	11937.83069	8.37641E-05	DLG4
SPN1 & Epilepsy_M1	2	2	0.066315789	30.15873016	0.001996751	CAMK2A, DLG4
SPN1 & ASD_ID_M2	2	1	0.044210526	22.61904762	0.04331216	DLG4
SPN2 & SZ_M1	2	1	0.095789474	10.43956044	0.091542197	MAPK8

Supplementary Table 12. Intersections among seven gene lists (SPN1, SPN2 plus five gene lists from Hormozdiari et al., Genome research 25, 142-154 (2015).

Gene	Sequences<br>(5' and 3' respectively)	NCBI reference	Amplicon size
Stat3	TCACTTGGGTGGAAAAGGAC<br>TGGTCGCATCCATGATCTTA	NM_011486	129
Hif1a	GAATGGAACGGAGCAAAAGA<br>CTGCCTTGTATGGGAGCATT	NM_001313919	138
Src	TCACCATCAAGTCGGATGTG<br>CACCTGGTCCAGAACCTCAC	NM_001025395	110
Dlg4	GCTCCCTGGAGAATGTGCT<br>TTCAAAGCTGTCGCCCTCTA	NM_001109752	137
Notch1	TGAACAATGTGGATGCTGCT<br>GCAACACTTTGGCAGTCTCA	NM_008714	131
Arnt2	TCACCAAGTGGAAACGCATA<br>CTCTGCTGTCCGTGATGCT	NM_007488	140
Ptgs2	TCATTCACCAGACAGATTGCT<br>AAGCGTTTGCGGTACTCATT	NM_011198.3	137
Socs3	CGTTGACAGTCTTCCGACAA<br>TATTCTGGGGGCGAGAAGAT	NM_007707.3	94
Lgals3	GATCACAATCATGGGCACAG<br>ATTGAAGCGGGGGTTAAAGT	NM_010705.3	100

Supplementary Table 13. qPCR primer sequences and their NCBI references, designed using Primer3 software.

**Axis a1**	**Axis a2**	**Axis a3**
x<10	x>=10 && x<=15	x>15
x<15	x>=15 && x<=20	x>20
x<20	x>=20 && x<=25	x>25
x<25	x>=25 && x<=30	x>30
x<30	x>=30 && x<=35	x>35
x<35	x>=35 && x<=40	x>40
x<40	x>=40 && x<=45	x>45
x<45	x>=45 && x<=50	x>50
x<50	x>=50 && x<=55	x>55
etc.	…	…
x<155	x>=155 && x<=160	x>160

Supplementary Table 14. Axis ranges used in animation in Supplementary Material 1.

Cohort	Clinical variable	mean with MA	mean without MA	Student ttest p-value
1	GA	28.59	28.43	0.79
1	PMA	39.09	38.28	0.56
2	GA	29.72	29.57	0.62
2	PMA	42.81	42.47	0.23
2	Days of ventilation	2.97	3.45	0.59

Cohort	Clinical variable	With MA [observed (expected)]	Without MA [observed (expected)]	Chi squared
2	Chorioamnionitis	9 (6.33)	4 (6.67)	0.129212481
2	Bacterial Infection	6 (5.85)	6 (6.15)	0.927048759
2	NEC surgery	2 (2.92)	4 (3.08)	0.446084346

Supplementary Table 15. Clinical features of infants in Cohort 1 and Cohort 2.

Supplementary Methods

Animal model

Experimental protocols were approved by the institutional guidelines of the Institut National de la Santé et de la Recherche Scientifique (Inserm, France), and met the guidelines for the United States Public Health Service's Policy on Humane Care and Use of Laboratory Animals (NIH, Bethesda, Maryland, USA). The experimental setup for inducing inflammation-induced white matter injury in the mouse has previously been described in detail [1]. In brief, a 5µL volume of phosphate-buffered saline (PBS) containing 10µg/kg injection of recombinant mouse IL1B or of PBS alone (control) was injected intra-peritoneally twice a day (morning and evening) on days postnatal P1 to P4 and once in the morning on day P5. Animals were sacrificed four hours after the morning injection of IL1B at P1, P5, P10 and P45. For microarray experiments, there were six biological replicates at each time-point which is considered to provide adequate statistical power [2-4], and all animals were male Swiss mice (OF1). All *in vivo* and *in vitro* experiments were performed using an alternating treatment allocation. All analyses were performed by an experimenter blinded to the treatment groups.

Fluorescence activated cell sorting in mouse

Dissociated cells from the cerebrum of mice pups (P1, P3, P5, P10) were centrifuged on a Percoll gradient, as previously described [5]. This protocol uses a digestion cocktail containing collagenase and dispase, and it involves separation over discontinuous percoll gradients. Cells were stained using different markers of myeloid cells. Neutrophils were defined as $CD11B^{hi}LY6G^{hi}$ whereas monocytes, macrophages and microglia are defined as $CD11B^{hi}LY6G^{lo}$. Monocytes were $F4/80^{lo}$ and microglia/macrophages were $F4/80^{hi}$. Finally, microglia were defined as $CD45^{lo}$ and macrophages as $CD45^{hi}$. Thus, cells were stained with anti-CD11B-PerCPCy5.5, LY6G-PE, CD45-FITC, F4/80-APC antibodies (BD Biosciences, NJ, USA). For analysis of purity of CD11B+ microglia MACSing (outlined below), only anti-CD11B-PerCPCy5.5, and F4/80-APC antibodies (BD Biosciences) were used because macrophages in the P1 brain of mice injected with PBS or IL1B represented only 0.6% and 1.7% respectively (Supplementary Figure 1). Cell suspensions were incubated with appropriate dilutions of fluorochrome-conjugated monoclonal antibodies and analysed on a fluorescence activated cell sorting (FACS) Calibur cytofluorimeter (BD Biosciences). Results were analysed with the Cell Quest Pro software (BD Biosciences). Absolute numbers of different cell populations were calculated by adding 10000 non-fluorescent 10µm polybead carboxylate microspheres (Polysciences, IL, USA) to each vial, and using the formula: Number of cells = (Number of acquired cells x 10,000)/(Number of acquired beads). Using

microspheres and percentages given by the software for each gate, the numbers of different cell populations can thus be obtained.

CD11B+ microglia magnetic activated cell sorting in mouse

Brains were collected from mice for cell dissociation, and microglia were isolated by magnetic antibody-based cell sorting (MACS) using CD11B antibody according to the manufacturer's protocol using all recommended reagents and equipment (Miltenyi Biotec, Bergisch Gladbach, Germany) and as previously described [6]. In brief, mice were intracardially perfused with NaCl 0,9%. After removing the cerebellum and olfactory bulbs, the brains were pooled (per sample at P1, n=3; at P5, n=2; at P10 & P45, n=1) and dissociated using the Neural Tissue Dissociation Kit containing papain enzyme and the gentleMACS Octo Dissociator with Heaters. Brain cells were enriched using the anti-CD11B (microglia) MicroBeads. After elution the isolated cells were centrifuged for 10 minutes at 300g and conserved at -80 °C until RNA extraction or placed into culture and treated as outlined for the primary microglia below. The purity of MACSed CD11B+ fractions was validated using FACS analysis of CD11B fluorescence (described above), and the purity was further validated with RT-qPCR of the positive and negative CD11B cell fractions. (Supplementary Figure 1). Specifically, we used RT-qPCR for glial fibrillary acid protein (Gfap), neuronal nuclear antigen (Neun), Myelin basic protein (Mbp) and Integrin Alpha M (Itgam) gene that encode CD11B. RT-qPCR was performed as described below and analysis confirmed that NeuN, Gfap and Mbp mRNA expression levels were extremely low in CD11B-possitive fraction compare to Itgam mRNA expression. Using CD11B-gated FACS analysis, we observed a slight but significant recruitment of peripheral immune cells - macrophages, monocytes and neutrophils - to the brain over time but no increase in the total number of microglia (Supplementary Figure 1, panel a,b). However, the relative contribution of these other immune cells to the total pool of CD11B+ cells was 100-1000 fold lower than that of microglia.

Blood brain barrier analysis in rat

The integrity of the blood brain barrier (BBB) was assessed following exposure to either 36 hours (3 injections) or 5 days (9 injections) of twice-daily intraperitoneal injections of IL1B (20µg/kg/injection) in the rat. The phenotype of the injury following IL1B mimics that observed in the mouse and was assessed as previously described, via gene expression analysis of the total brain and IgG staining of sections of the cerebral cortex [6-8]. Eight animals per group were used for the analysis. For gene expression analysis, pups were sacrificed by decapitation at P2 or P5, 5h after the last injection of IL1B. The brain was removed and the cerebrum was harvested and immediately frozen in liquid nitrogen. Total RNA was isolated using the Rneasy Mini Kit (Qiagen, Courtaboeuf, France) and qPCR performed as previously

described [6] (Methods). Genes validated for use as indicators of BBB breakdown were studied [7] and primers for each gene measured are listed in Supplementary Table 13. Data are reported as relative to the reference gene, *Gapdh*. For the analysis of IgG staining, rat pups were killed by decapitation at P5, 5h after the last injection of IL1B. The brains were fixed immediately in 4% formalin and post-fixed for 5 days. Following processing and paraffin embedding, sections were prepared at 10µm and immunolabelling with the antibody anti rat IgG (Sigma, B7139) was performed using the streptavidin-biotin-peroxidase method, as previously described [8].

Microarrays of mouse microglia gene expression and data pre processing

RNA was extracted and hybridised to Agilent Whole Mouse Genome Oligo Microarrays (8x60K). One biological replicate of IL1B exposure at P1 was removed from the analysis due to low RNA integrity, leaving a total number of 47 samples. Background-corrected $\log_2$ intensity data were quantile normalised and assigned detection p-values based on level of intensity and proximity to baseline. Genes with an expression p-value <0.05 across all samples were retained for analysis, resulting in a subset of roughly 23000 genes. Multivariate normality was confirmed with a test for kurtosis implemented in the R package ICS [9]. Assessment of variance of the data by multi-dimensional scaling (limma package in R [10]) confirmed similar distribution of samples across the IL1B and PBS groups.

Mouse microglia transcriptional response to IL1B and development

To assess responses gene by gene at each time-point, response to IL1B was assessed by subtracting mean expression values in control PBS from IL1B samples, using hierarchical clustering to group genes by similarity of normalised expression profile, implemented in the heatmap.2 tool in the R gplots package [11] (Figure 1, Panels b,c , Supplementary Data 5, Supplementary Figure 4). Clusters 1-4 were chosen based on magnitude of changes ($-1 \geq$ Z-score ≥ 1).

Multivariate analysis of variance (maanova R package [12]) was used to evaluate global effects of IL1B exposure and development on gene expression, including an interaction effect of both. Results were filtered to retain changes with p-value < 0.05 (corresponding to a false discovery rate (FDR) 10%[13]) and high coefficient of variation, resulting in three subsets of significantly expressed, highly varying, differentially expressed genes (Supplementary Data 1).

Gene co-expression network reconstruction from mouse microglia

Gene co-expression networks were inferred individually for the Development, IL1B and Interaction responses, using Graphical Gaussian Models implemented within the R software package "GeneNet" [14, 15]. This computes partial correlations, which are a measure of conditional independence between two genes i.e. the correlation between two genes after the common effects of all other genes are removed. Three separate gene networks were built from the sets of genes identified by MANOVA to show a significant response to IL1B, Development and Interaction effect (Figure 2). Local FDR was set at $1e^{-13}$ (the most stringent threshold possible, to minimise network size) and a minimum edge-wise partial correlation was set at 0.0075 (the point from which partial correlations increased exponentially across all networks). Hiveplots [16] were used to illustrate topological differences between the networks; the axes relate to ranges of node degree and nodes are plotted on each axis according to their degree. An illustrative hiveplot is in Figure 2, with an animation displaying the entire range in Supplementary Video 1, and parameters in Supplementary Table 14.

Protein-protein interactions and Power Graph Analysis

The nodes of all three gene networks (IL1B, Development, and Interaction) were aggregated into one list and used to investigate protein interactions, with both the Netvenn [17, 18] and DAPPLE [19] tools. The Netvenn tool was also used to perform a Power Graph Analysis (PGA)[20] on the protein interaction network, and two super-powernodes (SPNs) were identified by examining which sets of powernodes were directly interconnected (Figure 3, Supplementary Figure 6).

Functional annotation of gene co-expression and PPI networks

Gene Ontology annotation and enrichment analysis was carried out with the WebGestalt platform [21], always using as background the list from which the current set of interest was drawn to avoid inflation of significance e.g. all significantly expressed genes as background for MANOVA genes, and MANOVA genes as background for gene networks.

The REVIGO tool [22] was used to summarise GO terms based on semantic similarity measures, using an algorithm akin to hierarchical (agglomerative) clustering. Highly semantically similar GO terms were grouped as guided by the p-values supplied alongside the GO terms. This non-redundant GO term set was visualised using multidimensional scaling to render the subdivisions and the semantic relationships in the data. The disease-gene links used by the Gene Disease Association tool (GDA) [23] are assembled from the Genopedia compendium in the HuGE database of Human Genetic Epidemiology [24] and the OMIM database [25], which are collections of data retrieved from biomedical literature and do not provide cell or tissue specific annotations. While these disease-gene associations are not tissue specific, the relevance and usefulness of protein-protein interaction modules to tissue or

cell type specific transcriptional programs has been previously shown by our group and others [26-28].

Transcription factor analysis

The transcriptional control of the protein networks was interrogated in several independent ways. Transcription factor affinities for promoter sequences in the genes coding for the proteins of interest were predicted using the PASTAA tool [29]. Transcription binding sites for STAT1, STAT3 and STAT5 TFs were identified from experimental ChIP-Chip data of microglia in a P1 rat model with LPS exposure (peaks with FDR <0.2)[30]. Correlation of transcription factor expression levels and potential targets was calculated by finding the linear correlation between each transcription factor and each gene individually.

Primary ex vivo mouse microglia isolated by MACS

Primary microglia were prepared from the brain of P1 mice pups. Brain tissues were dissociated using the Neural Tissue Dissociation Kit containing papain and the gentleMACS Octo Dissociator with Heaters. Microglia were isolated using anti-CD11B (microglia) microbeads (MACS Technology), according to the manufacturer's protocol (Miltenyi Biotec, Germany) as described above for the preparation of microarray samples. CD11B+ microglia were pelleted via centrifugation and re-suspended in DMEM F12/PS/10% FBS at a concentration of $5x10^6$ cellsmL^{-1}. Cells were plated in 12-well plates (1ml/well) for RT-qPCR analysis. For immunofluorescence analysis, cells were plated in µ-Slide 8 Well Glass Bottom (Ibidi, Biovalley, France).

Treatment of mouse microglia with inhibitors for DLG4 and STAT3

Two days after plating, microglia were treated for 6 to 12 hours with vehicle (DMSO, 10µL/ml of culture media), IL1B at 50 ng/mL + IFNg at 20ng/ml, IL1B at 50 ng/mL + IFNg and 20ng/ml + Bp-1-102 (a STAT3 inhibitor; Merck Millipore, Fontenay sous Bois, France) [31] at 15 or 30 µM, or IL1B at 50 ng/mL + IFNg at 20ng/ml + TAT-N-dimer (an inhibitor of the DLG4 protein NMDA receptor interaction; Merck Millipore) at 15 or 30nM. Bp-1-102 and TAT-N-dimer were added one hour before the cytokines. At the end of the treatment period, cells were harvested and mRNA extracted for gene expression analysis, and supernatant were collected for nitrites/nitrates or cytokines/chemokines measurement.

RNA extraction and quantification of gene expression by real-time qPCR

Total RNA from primary microglial cell cultures was extracted with the RNeasy mini kit according to the manufacturer's instructions (Qiagen, Courtaboeuf, France). RNA quality and concentration were assessed by spectrophotometry with the Nanodrop™ apparatus

(Thermoscientific, Wilmington, DE, USA). Total RNA (1-2µg) was subjected to reverse transcription using the iScript™ cDNA synthesis kit (Bio-Rad, Marnes-la-Coquette, France). RT-qPCR was performed in duplicate for each sample using SYBR Green Supermix (Bio-Rad) for 40 cycles with a 2-step program (5 seconds of denaturation at 96°C and 10 seconds of annealing at 60°C). Amplification specificity was assessed with a melting curve analysis. Primers were designed using Primer3 software, and sequences and their NCBI references are given in Supplementary Table 13. The relative expression of genes of interest (GOI) was determined relative to expression of the reference gene, Glyceraldehyde 3-phosphate dehydrogenase (GAPDH). Analyses were performed with the Biorad CFX manager 2.1 software.

Multiplex cytokine and chemokine assay

Microglia media harvested at the end of the treatment was centrifuged briefly to remove particulates (300xg for 10 minutes). Cytokine and chemokine levels in the microglial media were measured using a Bio-plex 200 with a 96-well magnetic plate assay according to the manufacturer's instructions (Biorad laboratories, Marnes la Coquette, France). Cytokine and chemokine measured included IL-1α, IL1B, IL-2, IL-6, IL-10, IL-12 (p70), IL-13, G-CSF, GM-CSF, IFNg, TNFá, CXCL1 (KC), CCL2 (MCP-1) and CCL5 (RANTES). All samples were run in duplicate and data was analysed with the Bio-Plex Manager software.

Nitrites and nitrates assay

Microglia media harvested at the end of the treatment was centrifuged briefly to remove particulates (300xg for 10 minutes). Nitrite/nitrate content was measured using the nitrate/nitrite colorometric assay kit (Cayman Chemical, Ann Arbor, MI, USA) as directed.

Phagocytosis assay

Phagocytosis of fluorescently labelled *E Coli* particles by microglia was assessed using the pHrodo Red *E. coli* BioParticles Conjugate (Life Technologies) according to the manufacture's instructions. In brief, 50,000 primary microglial prepared as described above were plated in 48 well plates and after 12 hours of incubation with IL1B at 50 ng/mL + IFN-at 20ng/ml in the presence or absence of TAT-N-dimer at 30nM, medium was changed to serum free media containing the recommended suspension of bioparticles. Cells were incubated for five hours, before the particle-containing media was removed, washed twice with serum free media, incubated with a solution of trypan blue for 1 min to quench extracellular fluorescence and 1 ml of serum containing media added to each well. The absorbance of each well (including cell free, bead free and media free controls) was read. To adjust for cell density, following reading of the plate, the cells were used in an MTT assay.

Immunohistochemistry of mouse brain sections and isolated cells

Male mice (OF1 strain; Charles River) subjected to the IL1B induced white matter injury outlined above were deeply anesthetized with sodium pentobarbital 3 hours post IL1B or PBS injection at P1, P3 and P10 and perfused transcardially with 4% PFA in 0.1 M phosphate buffer. Brains were post-fixed in the same fixative solution for 2 hours or overnight at 4°C and cryoprotected with 30% sucrose in PBS at 4°C before inclusion in 7% gelatin, 15% sucrose in PBS and freezing in liquid isopentane at -50°C. 12μm thick coronal sections were cut on a cryostat, placed on glass slides and stored at -20°C until immunofluorescent labelling. Antibodies used were a mouse monoclonal antibody to detect DLG4 ((6G6-1C9) (Product# MA1-045), Thermo Scientific; 1:500[32]), a goat polyclonal antibody to detect IBA1 Ionized calcium binding adaptor molecule 1 ((IBA1) (ab5076, Abcam; 1:400 [33]), and a rabbit polyclonal antibody to detect Lysosomal-associated membrane protein 1 (LAMP-1) (L1418, Sigma ; 1/200). Secondary antibodies used were cyanine 3-conjugated donkey anti-mouse (Jackson ImmunoResearch Laboratories; 1:500), AlexaFluor-488-conjugated donkey anti-goat (Invitrogen; 1:500) and DyLight-405- conjugated donkey anti-rabbit (Jackson ImmunoResearch Laboratories; 1:500).

For sections mounted on glass slides were rehydrated in PBS and pre-incubated in PBS with 0.2% gelatin and 0.25% Triton X-100 (PBS-T-gelatin) for 15 minutes followed by overnight incubation with primary antibodies (anti-PSD95 and anti-IBA1) diluted in PBS-T-gelatin. The sections were rinsed with PBS-T-gelatin and incubated with secondary antibodies diluted in PBS-T-gelatin for 1.5 hours. Depending on the experiments, sections were then rinsed with PBS and incubated with DAPI diluted in PBS (1:1000) for 5 minutes for counter-staining of cell nuclei. All incubations were performed at room temperature, protected from light in a humidified chamber. Finally, the sections were rinsed with PBS, coverslipped with Fluoromount (Southern Biotech) and stored at 4°C until confocal microscopic analysis.

Microglial cells were permeabilized and blocked for 1 h using PBS/0.1% triton/3% horse serum (HS). Primary antibodies: goat anti-IBA1 (Abcam 1:500) and rabbit anti-DLG4 (PSD95) (Abcam, 1:500) or mouse anti-DLG4 (PSD95) (Thermofischer, 1:100), were applied overnight at 4 °C in PBS/1%HS. Fluorescently conjugated secondary antibody to rabbit IgG Cy3 and to goat IgG Alexa 488, were applied for 2 h at 20–25 °C in a humid chamber (1:500, Invitrogen). DAPI (1/500) was applied for 10 minutes (Figure 4, Supplementary Figure 12).

Human brain gene expression

Data on *DLG4* gene expression in the developing brain were accessed from the BrainCloud resource, which includes 30,176 probes on 269 samples across the lifespan (fetal through the aged)[34] and plotted (Supplementary Figure 15, panel a).

Developing Transcriptome data as described in the technical white paper were downloaded for *DLG4* [35] and plotted (Supplementary Figure 15, panel b). Expression of *DLG4* in the adult brain was visualized in the Allen Brain Atlas Brain Explorer software application (http://human.brain-map.org/static/brainexplorer), in which samples from the cerebral cortex are overlaid on an inflated white matter surfaces for each donor's brain, while samples in the subcortical regions of the brain are represented as spheres below the inflated surfaces.

Paired gene expression and genotype data from human brain were queried in the UKBEC collection of 134 brains from individuals free of neurodegenerative disorders [36]. Regional expression of *DLG4* (probeset mean) was extracted for all areas including white matter (Supplementary Figure 8, panel c). Genotypes for rs17203281 in all individuals were also accessed.

MACS isolation and inflammatory activation of human CD11B+ microglia

All human post-mortem tissue (cells and tissues) was acquired with ethical approval at The French Agency of Biomedicine (Agence de Biomédicine; approval PFS12-0011). Written informed consent was received prior to donation of fetal tissue. For the collection of human microglia, post-mortem tissue without any neuropathological alterations was acquired within 1 hour of scheduled termination (samples from two individuals, 19 gestational weeks (GW) and 21GW). Brain tissue (4g) was mechanically dissociated using 1ml micropipettor in HBSS with Ca^{2+} and Mg^{2+}, and a single cell suspension was obtained using a 70µM strainer. Isolated microglia were obtained using anti-CD11B microbeads (MACS Technology), according to the manufacturer's protocol (Miltenyi Biotec, Germany), as described above. CD11B+ microglia were pelleted via centrifugation and re-suspended in DMEM/PS/10% FBS at a concentration of $5x10^6$ cells/mL. Cells were plated in 12-well plates (1ml/well) for RT-qPCR analysis. For immunofluorescence analysis, cells were plated in µ-Slide 8 Well Glass Bottom (Ibidi, Biovalley, France).

Forty-eight hours after plating, human CD11B+ microglia were treated for 4 hours with DMEM (control) or lipopolysaccharide (LPS) 10 ng/mL diluted in DMEM. For RT-qPCR media were removed and plates frozen at -80°C. For immunofluorescence experiments, cells were fixed at room temperature with 4% paraformaldehyde for 20 minutes. Lipopolysaccharide (LPS) was used to stimulate human microglia in order to ensure an

inflammatory response, since the concentration of IL1B+IFNg used in the mouse experiments might not have been appropriate and we had too few cells available to perform a dose response. We had confidence that the high potency of LPS would lead to a pro-inflammatory response and this was confirmed by the increased expression of TNFa (data not shown).

Immunohistochemistry of developing human brain sections

Human brain sections were obtained from post mortem cases from medical abortions at 20GW, 26GW and 30GW for non-neurological diagnoses, under ethical approval at The French Agency of Biomedicine (Agence de Biomédicine; approval PFS12-0011). Tissue was fixed with 4% paraformaldehyde, frozen and sections cut at 12um. Staining for IBA1 (ab5076) and DLG4 (MA1-045) was performed as for mouse tissues above.

Immunohistochemistry of ex vivo human microglia and brain sections

For the collection of human microglia, post-mortem tissue without any neuropathological alterations was acquired within 1 hour of scheduled termination (samples from two individuals, 19 and 21 gestational weeks (GW), as described above. For the visualisation of IBA1 and DLG4 in human MACS isolated microglia ex vivo*,* slides were permeabilised and blocked for 1 h and primary antibodies, goat anti-IBA1 (ab5076, 1:500) and rabbit anti-PSD95 (MA1-045, 1:500) were applied overnight at 4°C. Fluorescently conjugated secondary antibodies from Invitrogene (1:1000) to rabbit IgG Cy3 and to goat IgG Alexa 488, were applied for 2 h at 20–25 °C in a humid chamber (1:500, Invitrogen).

Confocal microscopy

Immunofluorescent stainings of mouse and human tissues and cells were analysed using a Leica TCS SP8 confocal scanning system (Leica Microsystems) equipped with 488 nm Ar, 561 nm DPSS, and 633 nm HeNe lasers. Eight-bit digital images were collected from a single optical plane using a 63X HC PL APO CS2 oil-immersion Leica objective (numerical aperture 1.40). For each optical section, triple-fluorescence images were acquired in sequential mode to avoid potential contamination by linkage-specific fluorescence emission cross talk. Settings for laser intensity, beam expander, pinhole (1 Airy unit), range property of emission window, electronic zoom, gain and offset of photomultiplicator, field format, and scanning speed were optimized initially and held constant throughout the study so that all sections were digitized under the same conditions.

Imaging study patient characteristics

Cohort 1: Suitable MR images were acquired for 70 preterm infants at term-equivalent age (mean gestational age (GA) 28+4 weeks, mean postmenstrual age (PMA) at scan 40+3

weeks). The cohort consisted of preterm neonates who received care at Queen Charlotte's and Chelsea Hospital between January 2005 and October 2008, underwent DTI and MR imaging in the neonatal period. Infants were not eligible if they had a chromosomal abnormality, congenital malformation, or congenital infection.

Cohort 2: 271 infants (mean GA 29+4 weeks) had suitable imaging at term-equivalent age (mean PMA 42+4 weeks) as part of the EPRIME study (Evaluation of Magnetic Resonance (MR) Imaging to Predict Neurodevelopmental Impairment in Preterm Infants) and were imaged at term equivalent age over a 3 year period (2010-2013) at the Queen Charlotte and Chelsea Hospital, London. Research was carried out in compliance with the Code of Ethics of the World Medical Association (Declaration of Helsinki), with approval from the NHS National Research Ethics Service (NRES) and to the standard of the associated granting agencies. All MRI studies were supervised by an experienced paediatrician or nurse. Pulse oximetry, temperature, and heart rate were monitored throughout the period of image acquisition; ear protection in the form of silicone-based putty placed in the external ear (President Putty, Coltene; Whaledent) and Mini-muffs (Natus Medical Inc.) were used for each infant.

Image Acquisition

Cohort 1: Imaging was performed on a Philips 3-Tesla system (Philips Medical Systems, Netherlands) using an eight-channel phased array head coil. Single-shot echo-planar diffusion tensor imaging (EPI DTI) was acquired in the transverse plane in 15 noncollinear directions using the following parameters: repetition time (TR): 8000 ms; echo time (TE): 49 ms; slice thickness: 2 mm; field of view: 224 mm; matrix: 128 x 128 (voxel size: 1.7531 x 1.753 x 2 mm^3); b value: 750 smm^{-2}; SENSE factor: 2. For registration and clinical purposes, a T2-weighted fast-spin echo MRI was also acquired using: TR = 8700 ms, TE = 160 msec, flip angle = 90°, acquisition plane = axial, voxel size = 1.15 x 1.18 x 2 mm, FOV = 220 mm, and acquired matrix = 192 x 186.

Cohort 2: MRI was performed on a Philips 3-Tesla system (Philips Medical Systems, Netherlands) using an 8-channel phased array head coil. The 3D-MPRAGE and high-resolution T2-weighted fast spin echo images were obtained before diffusion tensor imaging. Single-shot EPI DTI was acquired in the transverse plane in 32 non-collinear directions using the following parameters: repetition time (TR): 8000 ms; echo time (TE): 49 ms; slice thickness: 2 mm; field of view: 224 mm; matrix: 128 × 128 (voxel size: 1.75 × 1.75 × 2 mm^3); *b* value: 750 smm^{-2}. Data were acquired with a SENSE factor of 2. T2-weighted fast-spin echo MRI was also acquired using TR = 8,670 ms, TE = 160 ms, flip angle = 90°, slice thickness = 2 mm, field of view = 220 mm, matrix = 256 × 256 (voxel size = 0.86 × 0.86 × 1 mm^3).

Imaging data selection and quality control

The T2-weighted MRI anatomical scans were reviewed in order to exclude subjects with extensive brain abnormalities, major focal destructive parenchymal lesions, multiple punctate white matter lesions or white matter cysts, since these infants represent a heterogeneous minority (1-3%) with different underlying biology and clinical features to the general preterm population [37-40]. All MR-images were assessed for the presence of image artefacts (inferior-temporal signal dropout, aliasing, field inhomogeneity, etc.) and severe motion (for head-motion criteria see below). All exclusion criteria were designed so as not to bias the study but preserve the full spectrum of clinical heterogeneity typical of a preterm born population.

Diffusion tensor imaging (DTI) analysis was performed using FMRIB's Diffusion Toolbox (FDT v2.0) as implemented in FMRIB's Software Library (FSL v4.1.5; www.fmrib.ox.ac.uk/fsl) [41]. Each infant's diffusion weighted images were registered to their non-diffusion weighted (*b*0) image and corrected for differences in spatial distortion due to eddy currents. Non-brain tissue was removed using the brain extraction tool (BET)[42]. FA maps were constructed from 15 or 32 direction DTI, and Tract Based Spatial Statistics [43] was used to obtain a group white matter skeleton by using a modified pipeline specifically optimized for neonatal DTI analysis [44]. Diffusion tensors were calculated voxel wise, using a simple least squares fit of the tensor model to the diffusion data. From this, the tensor eigenvalues and FA maps were calculated, and thresholded at FA > 0.2, then linearly adjusted for PMA at scan and GA.

Infant genotyping

Saliva samples for both infant cohorts were genotyped on the Illumina HumanOmniExpress-12 array, as previously described [45] (Supplementary Methods). The genotype matrix was recoded in terms of minor allele counts, including only SNPs with MAF ≥ 5% and ≥ 99% genotyping rate. Seven of these SNPs mapped to the *DLG4* region [46](Supplementary Table 10).

The concentration of all the genomic DNA samples was measured using the PicoGreen protocol. 200 ng of genomic DNA was used for each Illumina HumanOmniExpress-12 array according to the manufacturer's instructions. Huma-nOmniExpress-12 arrays have 730,525 markers with a mean spacing of ~4 kb. 392,197 of those markers are within 10 kb of a known RefSeq gene and there are 15,062 coding SNPs and 7459 MHC markers included in that total. All samples successfully passed quality control.

Association of imaging features with genotype

A general linear model was applied in FSL to test for association between FA values and minor allele count in Cohort 1, and significance was assessed using the randomise tool for nonparametric permutation inference on neuroimaging data [47] with threshold-free cluster enhancement (TFCE) inference [48] (Figure 6). This procedure was repeated independently for Cohort 2. There was no significant difference in clinical features (GA, PMA or days of ventilation) between infants with or without the minor allele in either Cohort (Supplementary Table 15).

***DLG4* expression quantitative trait loci analysis**

UKBEC genotype and expression data as described above were downloaded for *DLG4* white matter samples and SNP rs17203281. We used the transcript-level expression profile provided in the UKBEC dataset, which is estimated as the Windsorized mean (similar to trimmed mean) of the exon-level probesets that are considered expressed above background noise. It is chosen because it is robust to statistical outliers that may arise from alternative splicing.

Individuals were grouped according to minor allele count 0,1,2 or presence/absence of the minor allele (A) and outliers in the expression value were removed following thresholding by the boxplot.stats function in R. Significant group differences in expression values for the transcription-level were identified using a Student's t-test (Supplementary Figure 17).

SUPPLEMENTARY REFERENCES

1. Favrais, G., *et al.* Systemic inflammation disrupts the developmental program of white matter. *Annals of neurology* **70**, 550-565 (2011).
2. Illumina. The Power of Replicates. in *Technical Note: Gene Expression Profiling* (2010).
3. Pan, W., Lin, J. & Le, C.T. How many replicates of arrays are required to detect gene expression changes in microarray experiments? A mixture model approach. *Genome biology* **3**, research0022 (2002).
4. de Rinaldis, E.L., A. *DNA Microarrays: Current Applications* (Horizon Scientific Press, 2007).
5. Cardona, A.E., Huang, D., Sasse, M.E. & Ransohoff, R.M. Isolation of murine microglial cells for RNA analysis or flow cytometry. *Nat Protoc* **1**, 1947-1951 (2006).
6. Schang, A.L., *et al.* Failure of thyroid hormone treatment to prevent inflammation-induced white matter injury in the immature brain. *Brain, behavior, and immunity* **37**, 95-102 (2014).
7. Moretti, R., *et al.* Melatonin reduces excitotoxic blood-brain barrier breakdown in neonatal rats. *Neuroscience* **311**, 382-397 (2015).
8. Charriaut-Marlangue, C., *et al.* Sildenafil mediates blood-flow redistribution and neuroprotection after neonatal hypoxia-ischemia. *Stroke* **45**, 850-856 (2014).

9. Nordhausen, K.H., O.; Tyler, D.E. Tools for Exploring Multivariate Data: The Package ICS. *Journal of Statistical Software* **28** (2008).
10. Ritchie, M.E.*, et al.* limma powers differential expression analyses for RNA-sequencing and microarray studies. *Nucleic acids research* **43**, e47 (2015).
11. Warnes, G. & al., e. gplots: Various R Programming Tools for Plotting Data. (2015).
12. Wu, H., Yang, H., Churchill, G., Kerr, K. & Cui, X. maanova: Tools for analyzing Micro Array experiments. R package.
13. Storey, J.D. A direct approach to false discovery rates. *Journal of the Royal Statistical Society: Series B (Statistical Methodology)* **64**, 479-498 (2002).
14. Opgen-Rhein, R. & Strimmer, K. From correlation to causation networks: a simple approximate learning algorithm and its application to high-dimensional plant gene expression data. *BMC systems biology* **1**, 37 (2007).
15. Schafer, J. & Strimmer, K. An empirical Bayes approach to inferring large-scale gene association networks. *Bioinformatics* **21**, 754-764 (2005).
16. Krzywinski, M., Birol, I., Jones, S.J. & Marra, M.A. Hive plots--rational approach to visualizing networks. *Briefings in bioinformatics* **13**, 627-644 (2012).
17. Calderone, A., Castagnoli, L. & Cesareni, G. mentha: a resource for browsing integrated protein-interaction networks. *Nature methods* **10**, 690-691 (2013).
18. Wang, Y., Thilmony, R. & Gu, Y.Q. NetVenn: an integrated network analysis web platform for gene lists. *Nucleic acids research* **42**, W161-166 (2014).
19. Rossin, E.J.*, et al.* Proteins encoded in genomic regions associated with immune-mediated disease physically interact and suggest underlying biology. *PLoS genetics* **7**, e1001273 (2011).
20. Royer, L., Reimann, M., Andreopoulos, B. & Schroeder, M. Unraveling protein networks with power graph analysis. *PLoS computational biology* **4**, e1000108 (2008).
21. Wang, J., Duncan, D., Shi, Z. & Zhang, B. WEB-based GEne SeT AnaLysis Toolkit (WebGestalt): update 2013. *Nucleic acids research* **41**, W77-83 (2013).
22. Supek, F., Bosnjak, M., Skunca, N. & Smuc, T. REVIGO summarizes and visualizes long lists of gene ontology terms. *PloS one* **6**, e21800 (2011).
23. Park, J.*, et al.* Finding novel molecular connections between developmental processes and disease. *PLoS computational biology* **10**, e1003578 (2014).
24. Lin, B.K.*, et al.* Tracking the epidemiology of human genes in the literature: the HuGE Published Literature database. *Am J Epidemiol* **164**, 1-4 (2006).
25. McKusick, V.A. Mendelian Inheritance in Man and its online version, OMIM. *American journal of human genetics* **80**, 588-604 (2007).
26. Hoerder-Suabedissen, A.*, et al.* Expression profiling of mouse subplate reveals a dynamic gene network and disease association with autism and schizophrenia. *Proceedings of the National Academy of Sciences of the United States of America* **110**, 3555-3560 (2013).
27. Johnson, M.R.*, et al.* Systems genetics identifies Sestrin 3 as a regulator of a proconvulsant gene network in human epileptic hippocampus. *Nat Commun* **6**, 6031 (2015).
28. Delahaye-Duriez, A.*, et al.* Rare and common epilepsies converge on a shared gene regulatory network providing opportunities for novel antiepileptic drug discovery. *Genome biology* **17**, 245 (2016).
29. Roider, H.G., Manke, T., O'Keeffe, S., Vingron, M. & Haas, S.A. PASTAA: identifying transcription factors associated with sets of co-regulated genes. *Bioinformatics* **25**, 435-442 (2009).
30. Przanowski, P.*, et al.* The signal transducers Stat1 and Stat3 and their novel target Jmjd3 drive the expression of inflammatory genes in microglia. *Journal of molecular medicine* **92**, 239-254 (2014).
31. Zhang, X.*, et al.* Orally bioavailable small-molecule inhibitor of transcription factor Stat3 regresses human breast and lung cancer xenografts. *Proceedings of the National Academy of Sciences of the United States of America* **109**, 9623-9628 (2012).
32. Goddard, C.A., Butts, D.A. & Shatz, C.J. Regulation of CNS synapses by neuronal MHC class I. *Proceedings of the National Academy of Sciences of the United States of America* **104**, 6828-6833 (2007).

33. Low, P.C., *et al.* PI3Kdelta inhibition reduces TNF secretion and neuroinflammation in a mouse cerebral stroke model. *Nat Commun* **5**, 3450 (2014).
34. Colantuoni, C., *et al.* Temporal dynamics and genetic control of transcription in the human prefrontal cortex. *Nature* **478**, 519-523 (2011).
35. BrainSpan. TECHNICAL WHITE PAPER: TRANSCRIPTOME PROFILING BY RNA SEQUENCING AND EXON MICROARRAY. (2013).
36. Weale, M.E., *et al.* UK Brain Expression Consortium (UKBEC). (2012).
37. van Haastert, I.C., *et al.* Decreasing incidence and severity of cerebral palsy in prematurely born children. *J Pediatr* **159**, 86-91 e81 (2011).
38. Hamrick, S.E., *et al.* Trends in severe brain injury and neurodevelopmental outcome in premature newborn infants: the role of cystic periventricular leukomalacia. *J Pediatr* **145**, 593-599 (2004).
39. Volpe, J.J. Chapter 8: Hypoxic-ischemic encephalopathy; neuropathology and pathogenesis. . in *Neurology of the newborn* 347–399 (Saunders Elsevier, Philadelphia, 2008).
40. Ancel, P.Y., *et al.* Cerebral palsy among very preterm children in relation to gestational age and neonatal ultrasound abnormalities: the EPIPAGE cohort study. *Pediatrics* **117**, 828-835 (2006).
41. Smith, S.M., *et al.* Advances in functional and structural MR image analysis and implementation as FSL. *NeuroImage* **23 Suppl 1**, S208-219 (2004).
42. Smith, S.M. Fast robust automated brain extraction. *Hum Brain Mapp* **17**, 143-155 (2002).
43. Smith, S.M., *et al.* Tract-based spatial statistics: voxelwise analysis of multi-subject diffusion data. *NeuroImage* **31**, 1487-1505 (2006).
44. Ball, G., *et al.* An optimised tract-based spatial statistics protocol for neonates: applications to prematurity and chronic lung disease. *NeuroImage* **53**, 94-102 (2010).
45. Krishnan, M.L., *et al.* Possible relationship between common genetic variation and white matter development in a pilot study of preterm infants. *Brain Behav*, e00434 (2016).
46. Johnson, A.D., *et al.* SNAP: a web-based tool for identification and annotation of proxy SNPs using HapMap. *Bioinformatics* **24**, 2938-2939 (2008).
47. Winkler, A.M., Ridgway, G.R., Webster, M.A., Smith, S.M. & Nichols, T.E. Permutation inference for the general linear model. *NeuroImage* **92**, 381-397 (2014).
48. Smith, S.M. & Nichols, T.E. Threshold-free cluster enhancement: addressing problems of smoothing, threshold dependence and localisation in cluster inference. *NeuroImage* **44**, 83-98 (2009).
